# Integrated biomarker profiling of the metabolome associated with impaired fasting glucose and type 2 diabetes mellitus in large‐scale Chinese patients

**DOI:** 10.1002/ctm2.432

**Published:** 2021-06-01

**Authors:** Jianglan Long, Hui Yang, Zhirui Yang, Qingquan Jia, Liwei Liu, Lingwei Kong, Huijing Cui, Suying Ding, Qian Qin, Nana Zhang, Xingzhong Feng, Shuxun Yan, Jinfa Tang, Shuo Chen, Yumei Han, Tao Jiang, Zhen Wen, Ningning Qi, Kejun Deng, Zhi Sun, Hao Lin, Dan Yan

**Affiliations:** ^1^ Beijing Friendship Hospital, Capital Medical University Beijing China; ^2^ Beijing Key Laboratory for Evaluation of Rational Drug Use Beijing China; ^3^ College of Life Science and Technology University of Electronic Science and Technology of China Chengdu Sichuan China; ^4^ Zhengzhou University First Affiliated Hospital Zhengzhou Henan China; ^5^ Beijing Jiaotong University Community Health Center Beijing China; ^6^ Department of Pharmacy Kaifeng Hospital of Traditional Chinese Medicine Kaifeng Henan China; ^7^ Department of Endocrinology and Immunology Yuquan Hospital of Tsinghua University Beijing China; ^8^ Department of Endocrinology The First Affiliated Hospital of Henan University of CM Zhengzhou Henan China; ^9^ Beijing Physical Examination Center Beijing China; ^10^ Beijing Shijitan Hospital, Capital Medical University Beijing China

Dear Editor,

Type 2 diabetes mellitus (T2DM) is an important cause of diabetes complications and mortality.^1^ The prevalence of prediabetes including impaired fasting glucose (IFG) is approximately one‐third of the population in China,^2^ but comprehensive early risk evaluation is weak. Thus, it is important to identify diagnostic biomarkers of prediabetes and T2DM and improve the disease risk prediction ability. We created the validated integrated biomarker profiling (IBP) related to the development of IFG and T2DM. Moreover, the established service website of the IBP implied potential clinical application.

To construct the IBPs of IFG (fasting blood glucose (FBG), 6.1 ≤ FBG < 7.0 mmol/L) and T2DM (FBG ≥ 7.0 mmol/L), 1705 participants (BMI < 30) from five centers in China recruited randomly assigned into the discovery (*n = *153), test (*n = *420), and validation phases (*n = *1132, 146 hyperlipidemia patients as an interference group, training set of 792 [69.96%], test set of 340 [30.04%]) (Figures 1A and B), which were homogeneous (Table 1).

**FIGURE 1:**
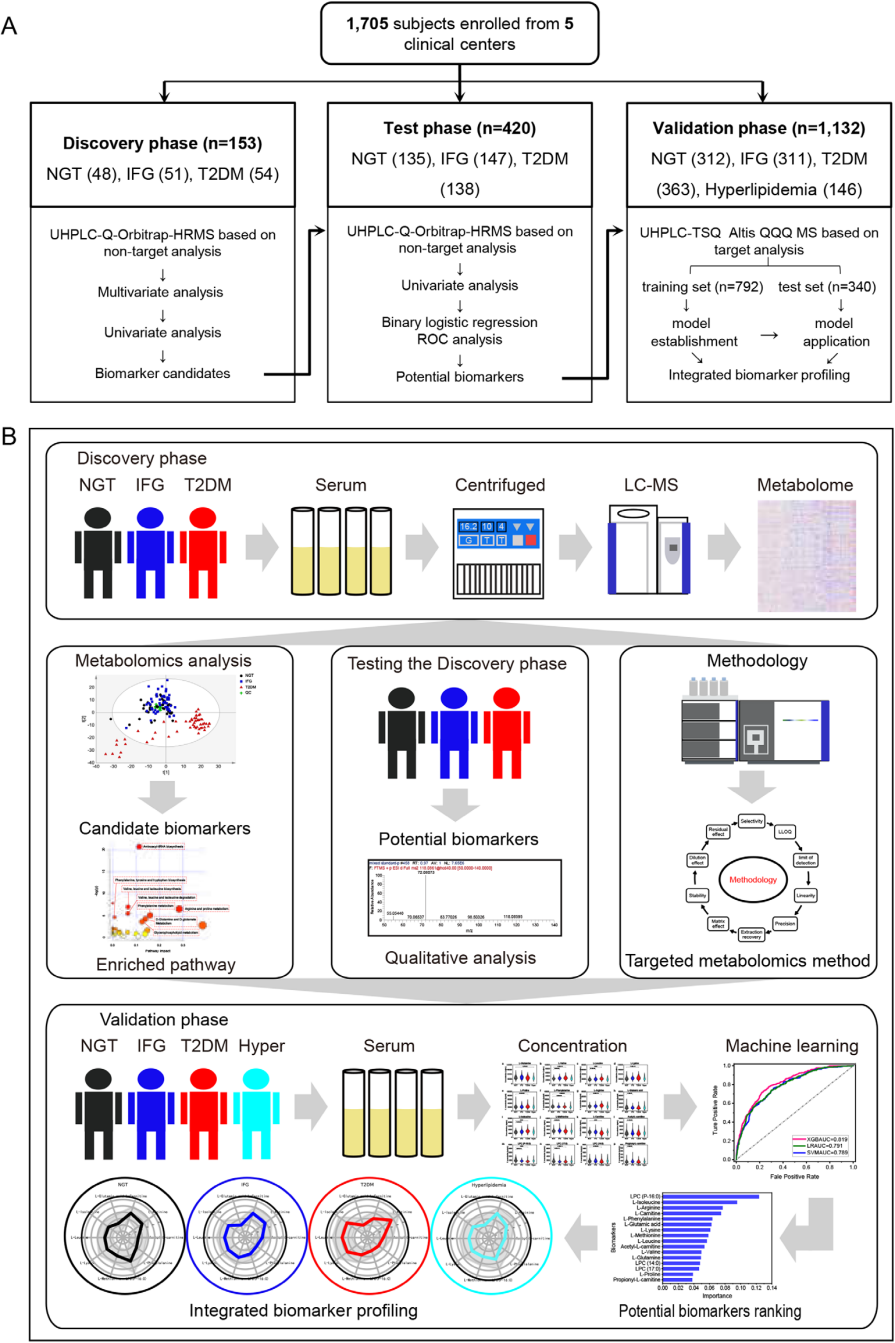
Study design. (A) The three‐step analysis strategy. Nontargeted metabolomics in the discovery and test phases was performed to identify and validate potential biomarkers. In the validation phase, the potential biomarkers were screened using Gini impurity to construct the integrated biomarker profilings of IFG and T2DM based on the eXtreme Gradient Boosting model, and individuals with hyperlipidemia were set as an interference group to evaluate the prediction accuracy of the integrated biomarker profiling. (B) The overview of study design. In the discovery phase, 153 subjects were enrolled to screen biomarker candidates of IFG and T2DM; 420 subjects were recruited to test the biomarker candidates in the test phase; in the validation phase, an independent training set of 792 subjects was used to construct the IBP prediction model for NGT, IFG, T2DM, and hyperlipidemia. Then, the IBP prediction models were evaluated with a test set of 340 subjects. Abbreviations: IFG, impaired fasting glucose; LLOQ, low limit of quantification; LPC, lysophosphatidylcholine; NGT, normal glucose tolerance; ROC, receiver operating characteristic curve; T2DM, type 2 diabetes mellitus; UHPLC‐Q‐Orbitrap‐HRMS, ultra‐high performance liquid chromatography Q Exactive‐Orbitrap high‐resolution mass spectrometer; UHPLC‐TSQ‐Altis QQQ MS, ultra‐high performance liquid chromatography TSQ Altis triple quadrupole mass spectrometer

**TABLE 1:** Clinic characteristics of the subjects

							Validation phase (*n = *1,132)							
	Discovery phase (*n = *153)			Test phase (*n = *420)			Training set (*n = *792)				Test set (*n = *340)			
	NGT	IFG	T2DM	NGT	IFG	T2DM	NGT	IFG	T2DM	Hyperlipidemia	NGT	IFG	T2DM	Hyperlipidemia
***n***	48	51	54	135	147	138	232	214	230	96	80	97	113	50
**Male (%)**	27 (56.25)	27 (52.94)	29 (53.70)	81 (60)	83 (56.56)	79 (57.25)	150 (64.66)	120 (56.07)	143 (57.2)	51 (53.13)	44 (55)	49 (50.52)	59 (52.2)	32 (64)
**Female (%)**	21 (43.75)	24 (47.06)	25 (46.30)	54 (40)	64 (43.54)	59 (42.75)	82 (35.34)	94 (43.93)	107 (42.8)	45 (46.87)	36 (45)	48 (49.48)	54 (47.8)	18 (36)
**Age (years)**	49.10 (10.58)	48.80 (10.45)	50.43 (9.86)	50.86 (9.57)	51.39 (9.82)	51.77 (9.89)	51.56 (9.69)	55.03 (9.58)	53.52 (9.51)	52.91 (10.97)	51.93 (10.06)	52.54 (9.82)	54.86 (10.08)	51.7 (11.31)
**FBG (mmol/L)**	5.42 (0.28)	6.40 (0.21)	9.74 (3.05)	5.49 (0.39)	6.44 (0.24)	10.93 (3.32)	5.36 (0.47)	6.46 (0.25)	11.37 (3.34)	5.48 (0.30)	5.40 (0.46)	6.41 (0.24)	10.89 (3.20)	5.51 (0.34)
**BMI (kg/m^2^)**	23.48 (2.91)	24.67 (3.35)	25.82 (3.88)	23.03 (2.70)	25.26 (3.00)	25.94 (2.75)	23.61 (2.60)	25.07 (2.71)	26.07 (2.89)	25.04 (3.24)	22.99 (2.75)	25.53 (3.53)	26.17 (3.04)	24.43 (2.73)
**LDL‐C (mmol/L)**	2.45 (0.39)	3.02 (0.70)	3.09 (0.83)	2.33 (0.44)	3.08 (0.81)	3.48 (0.98)	2.49 (0.46)	2.93 (0.84)	3.17 (0.92)	3.74 (0.61)	2.53 (0.45)	3.09 (0.84)	3.14 (0.88)	3.65 (0.64)
**HDL‐C (mmol/L)**	1.55 (0.30)	1.54 (0.34)	1.42 (0.40)	1.53 (0.29)	1.51 (0.34)	1.11 (0.27)	1.44 (0.26)	1.49 (0.31)	1.18 (0.27)	1.51 (0.31)	1.51 (0.29)	1.51 (0.32)	1.18 (0.28)	1.37 (0.31)
**TG (mmol/L)**	1.06 (0.26)	1.80 (1.11)	2.44 (1.65)	1.02 (0.31)	1.81 (1.31)	2.15 (1.88)	1.07 (0.32)	1.83 (1.41)	2.32 (2.42)	1.89 (0.88)	0.98 (0.31)	1.87 (1.60)	2.17 (2.38)	2.38 (1.58)
**TC (mmol/L)**	4.44 (0.48)	5.15 (0.91)	5.23 (1.06)	4.27 (0.54)	5.21 (1.00)	5.46 (1.23)	4.34 (0.51)	5.04 (1.05)	5.09 (1.23)	5.96 (0.77)	4.46 (0.50)	5.24 (1.00)	5.06 (1.32)	5.82 (0.77)
**ALT (U/L)**	17.04 (7.30)	26.27 (18.05)	29.19 (17.22)	16.70 (6.41)	25.22 (13.79)	29.00 (13.57)	17.16 (6.59)	22.58 (10.23)	26.87 (14.96)	30.43 (24.85)	18.30 (6.38)	23.85 (12.43)	30.5 (14.46)	33.32 (33.91)
**AST (U/L)**	19.13 (4.21)	22.67 (9.01)	21.85 (8.43)	19.57 (4.15)	21.58 (6.72)	27.62 (12.25)	18.97 (4.34)	20.95 (6.08)	25.41 (12.47)	25.58 (15.99)	21.26 (6.56)	22.25 (8.73)	26.96 (11.28)	23.8 (10.46)

Data are n (%) or mean (SD).

Abbreviations: ALT, alanine transaminase; AST, aspartate transaminase; BMI, body mass index; FBG, fasting blood glucose; HDL‐C, high‐density lipoprotein cholesterol; IFG, impaired fasting glucose; LDL‐C, low‐density lipoprotein cholesterol; NGT, normal glucose tolerance; TC, total cholesterol; TG, triglyceride; T2DM, type 2 diabetes mellitus.

The nontargeted metabolomics analysis was performed in the discovery and test phases to identify potential biomarkers of IFG and T2DM; in the validation phase, the potential biomarkers were quantified based on targeted metabolomics. In the discovery, after peak pretreatment using the 80% rule (Figures S1A‐S1G in Supporting Information),^3^ the quality control samples clustered together (Figures S2A‐S2B), which indicated that the analysis was stable and reliable. Furthermore, there were significant differences in metabolites among the normal glucose tolerance (NGT), IFG, and T2DM groups (Figures S2A‐S2F) with not overfitting (Figure S1H). After screening and identification, 31 and 42 biomarker candidates were identified in the fasted serum of IFG and T2DM patients, respectively (Tables S1‐S2 in Supporting Information). In the test phase, *P *< .05 was regarded as significant within similar retention time ranges. Basis on the results of logistic regression (LR) and receiver operating characteristic curve (ROC) analysis (Tables S3‐S4), 41 metabolites were regarded as the potential biomarkers of IFG or T2DM in multicenter (Table S5).

In the validation phase, the significant differences were found in the serum concentrations of the potential biomarkers (Figure S3). The results of risk analysis showed that l‐valine, l‐leucine, and l‐isoleucine appeared to be risk factors for IFG and T2DM; lysophosphatidylcholine (LPC, P‐16:0) appeared to be associated with a lower risk of IFG and T2DM, which was consistent with the previous studies.^4^, ^5^, ^6^ Oppositely, the concentration of l‐phenylalanine was lower in the serum of IFG and T2DM patients than in that of individuals with NGT, which was contrary to the reported findings (Table S6).^4^ Unfortunately, ROC analysis showed that the single potential biomarker performed poorly for the diagnosis of NGT, IFG, T2DM, and hyperlipidemia (Figure S4). Therefore, it is necessary to integrate multiple biomarkers to comprehensively reflect the occurrence and development of diabetes.

The differences in metabolites might mediate the occurrence and development of diabetes associated with insulin resistance and dysfunction of pancreatic islet β‐cells,^7^ which was manifested the disorder of glycerophospholipid metabolism and amino acid metabolic pathways (Figure S5). With the Krebs cycle as the hub,^4^, ^5^, ^7^ there was an interrelation among the metabolic pathways (Figure 2). Thus, the risk biomarkers of diabetes should be regarded as a whole biological event associated with biological network. Therefore, the construction of IBP to assess diabetes risk from the perspective of multiple biomarkers that related to the occurrence and development of IFG and T2DM provides a potential biological mechanism basis.^8^

**FIGURE 2:**
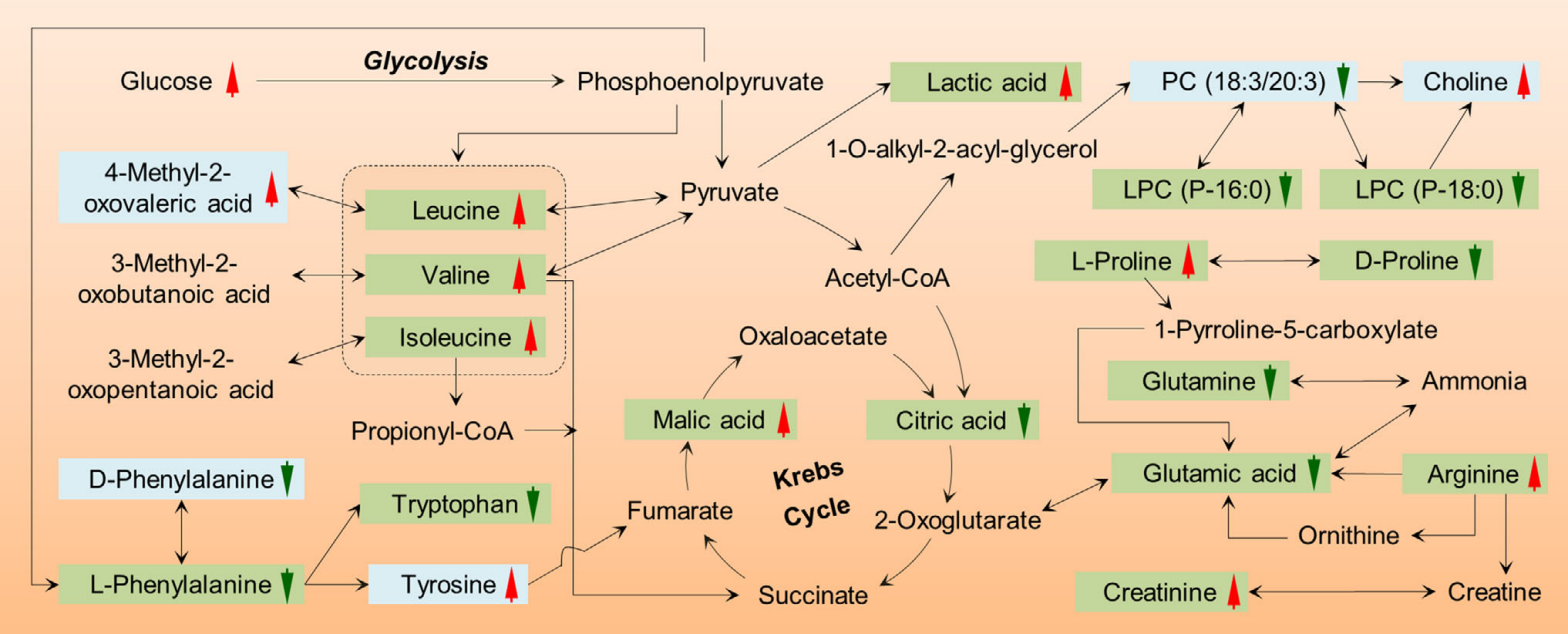
Coarse pictorial diagram of main metabolic pathways of candidate biomarkers and their interrelationships. Red arrows represent the notable increase, whereas green arrows represent the notable decrease of serum biomarkers (candidate biomarkers (aquamarine boxes) and potential biomarkers (grass boxes)). Black arrows represent one or more steps of an enzymatic reaction. Abbreviations: LPC, lysophosphatidylcholine; PC, phosphatidylcholine

**FIGURE 3:**
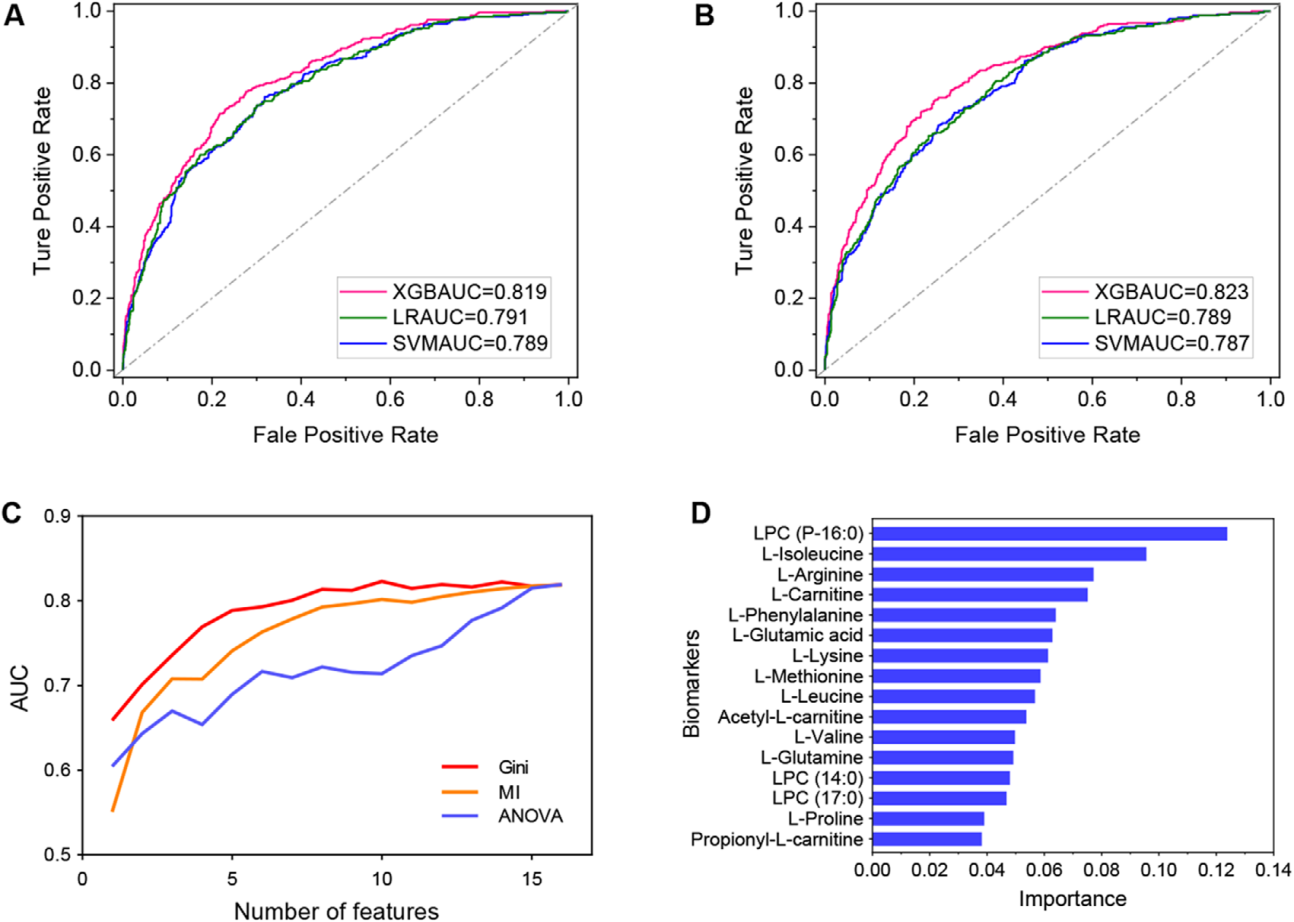
Establishment of integrated biomarker profiling. (A) AUC of the integrated 16 potential biomarkers from the LR, SVM, and XGBoost model. (B) AUC of the integrated 10 potential biomarkers from the LR, SVM, and XGBoost model. (C) Incremental feature selection curve of the integrated 16 potential biomarkers from analysis of variance, mutual information, and Gini impurity based on XGBoost model. (D) Gini impurity of 16 potential biomarkers. Abbreviations: AUC, area under the curve; LR, logistic regression; LPC, lysophosphatidylcholine; MI, mutual information; SVM, support vector machine; XGB, eXtreme gradient boosting

Simply considering multiple nonquantitative biomarkers as a single biomarker ignored the overlap of individuals with and without the incidence of disease, which compromised their discriminatory ability.^9^ Three machine‐learning methods (eXtreme Gradient Boosting [XGBoost], LR, and support vector machine [SVM]) were selected to construct prediction model. The results showed that the XGBoost model has better predicted performance (XGBAUC = 0.819, LRAUC = 0.791, and SVMAUC = 0.789; Figure 3A).^10^ Further, Gini impurity was used to select 10 biomarkers that have better prediction ability of IFG and T2DM disease risk from targeted 16 potential biomarkers to construct IBP (XGBAUC = 0.823, Figures 3B and C), which consisted of LPC (P‐16:0), l‐isoleucine, l‐arginine, l‐carnitine, l‐phenylalanine, l‐glutamic acid, l‐lysine, l‐methionine, l‐leucine, and acetyl‐l‐carnitine (Figure 3D).

The predicted performance of the IBP was satisfactory. In the discovery phase, the prediction accuracy of the IBP for discrimination of NGT, IFG, and T2DM was 96% with high sensitivity and specificity. The AUC values of the IBP in the discrimination of IFG and NGT, T2DM and NGT, T2DM and IFG, and T2DM and hyperlipidemia were 0.804, 0.936, 0.823, and 0.937, respectively (Figure S6). Moreover, the AUC value of the IBP for discrimination of NGT, IFG, and T2DM was 0.828 in the test phase (Table S7). The IBP showed that the concentrations of 10 potential biomarkers were different in NGT, IFG, and T2DM (Figure 4). An unknown and random sample might belong to the group that has the highest predictive value in NGT, IFG, T2DM, and hyperlipidemia groups based on the XGBoost model. For examples, if the predictive values of unknown sample 1 in NGT, IFG, T2DM, and hyperlipidemia group were 0.092, 0.676, 0.139, and 0.093, respectively (Figure 4D), which implied that it might be a patient with IFG (Figure 4C). The predictions for representative samples in the other groups were shown in Figure 4. Moreover, we established a website of the IBPs for IFG and T2DM for the first time (http://pdm.lin‐group.cn/) that can further improve the potential clinical public service ability of this study.

**FIGURE 4:**
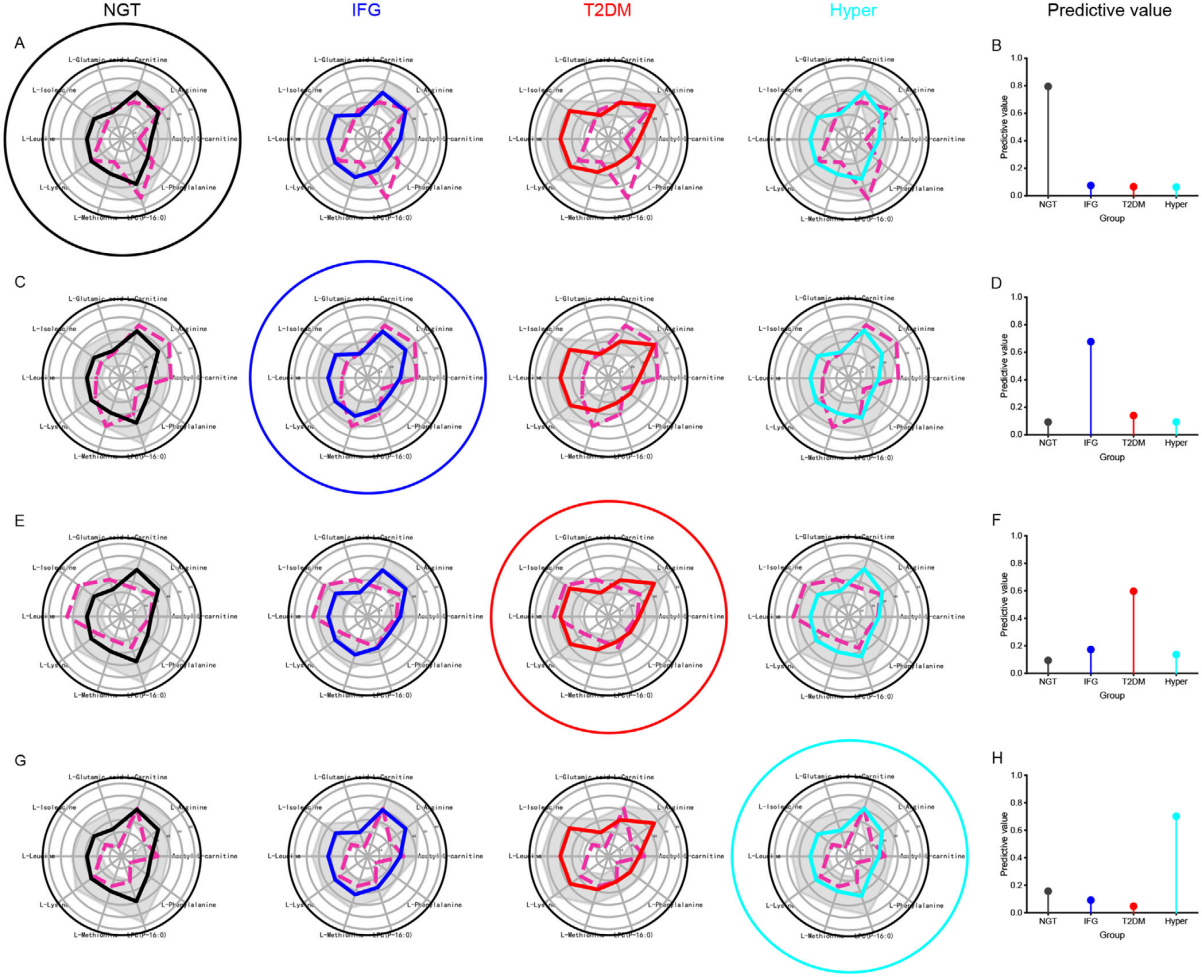
Schematic prediction diagram of several typical representative samples using the integrated biomarker profiling. Schematics for representative NGT (A) (the predictive values of unknown sample 1 in NGT, IFG, T2DM, and hyperlipidemia were 0.795, 0.075, 0.066, and 0.064, respectively), IFG (C) (0.092, 0.676, 0.139, and 0.093), T2DM (E) (0.094, 0.173, 0.597, and 0.137), and hyperlipidemia (G) (0.157, 0.092, 0.048, and 0.702) samples using the integrated biomarker profiling. Predictive values for representative samples belonging to the NGT (B), IFG (D), T2DM (F), and hyperlipidemia (H) groups. It could be interpreted that the unknown sample belongs to the group that has the highest predictive value. After normalized concentrations, solid line: the mean value of potential biomarkers in four groups; gray area: mean ± SD; dotted line: the normalized value of concentration of the potential biomarker in an unknown sample. Abbreviations: Hyper, hyperlipidemia; IFG, impaired fasting glucose; LPC, lysophosphatidylcholine; NGT, normal glucose tolerance; T2DM, type 2 diabetes mellitus

In conclusion, based on large sample data from the clinical real world, through metabolomics and machine learning methods, we have established the IBPs of IFG and T2DM and its public service website related to the occurrence and development of prediabetes and T2DM, which could avoid the use of multiple biomarkers that could confuse the interpretation of the results, reduce the impact of information fluctuation of single or isolated biomarker on the overall evaluation efficiency, and improve the auxiliary evaluation ability of the potential biomarkers for clinical diseases.

## ETHICS APPROVAL AND CONSENT TO PARTICIPATE

The study was approved by the Scientific Research Ethics Committee of Capital Medical University Affiliated Beijing Shijitan Hospital (2017‐035) and registered on the Chinese Clinical Trial Registry (ChiCTR1800014301). All the participants provided their written informed consent.

## CONSENT FOR PUBLICATION

Contents for publication were obtained from all patients.

## CONFLICT OF INTEREST STATEMENT

The authors declare that they have no competing interests.

## DATA AVAILABILITY STATEMENT

The data that support the findings of this study are available from the corresponding author upon reasonable request.

## AUTHOR CONTRIBUTIONS

DY conceived and proposed the research topic. JL and ZY designed the study protocol, which was edited by DY. JL and ZY collected samples. LK, HC, SD, QQ, NZ, XF, SY, JT, SC, YH, TJ, ZW, NQ, and KD contributed to sample collection. JL, QJ, LL, and ZS performed sample detection. HY and HL created the machine learning‐based classification algorithm. JL, HY, ZS, and DY participated in statistical analysis. JL, ZY, HY, HL, and DY contributed to writing, revising, and proofreading the manuscript. All authors read and approved the final manuscript.

## Supporting information

Supporting Information
