# Supplementary material for: Integrated biomarker profiling of the metabolome associated with impaired fasting glucose and type 2 diabetes mellitus in large‐scale Chinese patients
Source: Clin Transl Med. 2021 Jun 1;11(6):e432. doi: 10.1002/ctm2.432 (PMC8167862; doi:10.1002/ctm2.432)
Supplement: Supplementary file 1 — Supporting Information [file CTM2-11-e432-s001.doc]

**Supporting Information**

**Integrated biomarker profiling of the metabolome associated with impaired fasting glucose and type 2 diabetes mellitus in large-scale Chinese patients**

**Short running title: Integrated biomarker profiling of diabetes**

Jianglan Long^1,2,10#^, Hui Yang^3#^, Zhirui Yang^1,10#^, Qingquan Jia^4^, Liwei Liu^4^, Lingwei Kong^5^, Huijing Cui^5^, Suying Ding^4^, Qian Qin^4^, Nana Zhang^6^, Xingzhong Feng^7^, Shuxun Yan^8^, Jinfa Tang^8^, Shuo Chen^9^, Yumei Han^9^, Tao Jiang^10^, Zhen Wen^10^, Ningning Qi^10^, Kejun Deng^3^, Zhi Sun^4*^, Hao Lin^3*^, Dan Yan^1,2*^

^1^ Beijing Friendship Hospital, Capital Medical University, Beijing, China

^2^ Beijing Key Laboratory for Evaluation of Rational Drug Use, Beijing, China

^3^ College of Life Science and Technology, University of Electronic Science and Technology of China, Chengdu, Sichuan, China

^4^ Zhengzhou University First Affiliated Hospital, Zhengzhou, Henan, China

^5^ Beijing Jiaotong University Community Health Center, Beijing, China

^6^ Department of Pharmacy, Kaifeng Hospital of Traditional Chinese Medicine, Kaifeng, Henan, China

^7^ Department of Endocrinology and Immunology, Yuquan Hospital of Tsinghua University, Beijing, China

^8^ Department of Endocrinology, The First Affiliated Hospital of Henan University of CM, Zhengzhou, Henan, China

^9^ Beijing Physical Examination Center, Beijing, China

^10^ Beijing Shijitan Hospital, Capital Medical University, Beijing, China

^#^ Jianglan Long, Hui Yang, Zhirui Yang contributed equally to this study.

^*^ Corresponding authors: Dr. Zhi Sun, Zhengzhou University First Affiliated Hospital, No. 1, Jianshe East Road, Erqi District, Zhengzhou, Henan, 450052, China. Email: [fccsunz@zzu.edu.cn](mailto:fccsunz@zzu.edu.cn);

Prof Hao Lin, University of Electronic Science and Technology of China, No. 2006, Xiyuan Avenue, Gaoxin District, Chengdu, Sichuan, 610054, China. Email: [hlin@uestc.edu.cn](mailto:hlin@uestc.edu.cn);

Prof Dan Yan, Capital Medical University Affiliated Beijing Friendship Hospital, No. 95, Yong’an Road, Xicheng District, Beijing, 100050, China. Email: [danyan@ccmu.edu.cn](mailto:danyan@ccmu.edu.cn).

## Supplementary Materials

## 1. Study design and population

A total of 1,705 individuals with normal glucose tolerance (NGT), impaired fasting glucose (IFG), type 2 diabetes mellitus (T2DM), and hyperlipidemia (forming an interference group) were recruited at five clinical centers in China between January and June 2018, which randomly assigned into the discovery, test, and validation phases (Figures 1A−1B). In the discovery phase, to screen biomarker candidates, 153 individuals were recruited in Beijing Jiaotong University Community Health Center (Beijing, China). The test phase, involving 420 samples that used to test the biomarker candidates screened, took place at Beijing Jiaotong University Community Health Center, Beijing Physical Examination Center (Beijing, China), and Kaifeng Hospital of Traditional Chinese Medicine (Kaifeng, China). In the validation phase, to establish the integrated biomarker profiling (IBP) for IFG and T2DM, 1,132 samples from multicenter were used. The participants were from Beijing Jiaotong University Community Health Center, Beijing Physical Examination Center, Capital Medical University Affiliated Beijing Shijitan Hospital, Kaifeng Hospital of Traditional Chinese Medicine, and the First Affiliated Hospital of Henan University of Chinese Medicine (Zhengzhou, China).

The participants were placed into three groups according to the World Health Organization’s diagnosis and classification of diabetes,^1^ based on their fasting blood glucose (FBG) concentration: NGT individuals (3.9≤ FBG <6.1 mmol/L), individuals with IFG (6.1≤ FBG <7.0 mmol/L), and T2DM patients (FBG ≥7.0 mmol/L). Furthermore, a hyperlipidemia group (146 hyperlipidemia patients) was set as the interference group in the validation phase, which consisted of individuals with NGT but hyperlipidemia (triglyceride ≥1.7 mmol/L and/or total cholesterol ≥5.2 mmol/L).^2^ The exclusion criteria were participant with taken drugs in the past one-month, diabetes complications, liver disease or any other type of malignant disease for the T2DM patients, and a history of metabolic or other systemic disease for the healthy controls.

## 2. Metabolomics analysis

***2.1 Sample collection and pretreatment***

Blood samples were collected at 07:00–08:30 after an overnight fast. The samples were centrifuged at 1,510 × g for 10 min to obtain sera, which were immediately placed in a −80°C freezer (Thermo Fisher Scientific, San Jose, USA) for storage.

For the non-targeted metabolomics analysis, the serum samples were thawed on ice for 30 min,^3^ then 50 μL of each were mixed with 150 μL methanol solution (containing 50 ng/mL 2-chloro-L-phenylalanine (J&K Chemical, Beijing, China) and 500 ng/mL ketoprofen (Sigma-Aldrich, Missouri, USA) as the internal standards). After vortexing for 60 sec, the mixture was centrifuged at 16,200 × g and 4°C for 10 min to precipitate the protein. Then, 100 μL supernatant was transferred to an auto-sampler vial (Waters, Milford, USA) for analysis in positive and negative electrospray ionization (ESI^+^ and ESI^–^) modes, respectively. Partial samples of each group were selected randomly, then 10 μL of each were mixed to form mixed serum as the quality control (QC) sample, which could monitor the stability of the large-scale analysis.^4^ Pretreatment of the QC samples was paralleled to the analytical samples with the same treatment method.

For the targeted metabolomics analysis, 10 μL of each serum sample was diluted with 90 μL phosphate buffered saline.^5,6^ After vortexing for 60 sec, the diluted serum was mixed with 300 μL acetonitrile methanol solution (3:1, v:v) containing isotope internal standards (10 μg/mL Cell Free Amino Acid Mix (20 AA), 500 ng/mL O-acetyl-L-carnitine (N-methyl-D3) (acetyl-L-carnitine-d3) and 25 ng/mL lysophosphatidylcholine (20:0) (Eicosanoyl-12,12,13,13-D4) [LPC (20:0)-d4)]). After vortexing for 5 min, the mixture was centrifuged at 16,200 × g at 4°C for 10 min to precipitate the protein. Then, 200 μL supernatant was transferred to an auto-sampler vial for analysis in ESI^+^ mode.

***2.2 Non-targeted metabolomics analysis***

The non-targeted metabolomics analysis was performed in the discovery and test phases using a Dionex Ultimate 3000 UHPLC (ultra-high performance liquid chromatography) system coupled to a Q Exactive-Orbitrap high-resolution mass spectrometer (UHPLC-Q-Orbitrap-HRMS) (Thermo Fisher Scientific), which was equipped with a heat electrospray ionization system. In the sequence of sample analysis, QC samples were inserted into every ten samples to monitor the stability of the system.

**UHPLC separation**. A Dionex Ultimate 3000 ultra-high performance liquid chromatography (UHPLC) system (Thermo Fisher Scientific, San Jose, USA) equipped with a cooling auto-sampler, column oven, and UV detector was utilized. An ACQUITY BEH C18 column (100 mm × 2.1 mm, 1.7 μm) (Waters, Milford, USA) was employed at a temperature of 40°C. Mobile phases A and B were 0.1% formic acid aqueous solution and acetonitrile, respectively. The elution gradient started with 5% B and held for 1.0 min, then increased linearly to 100% B at 9.0 min. After held for 3.0 min with 100% B, the elution gradient decreased to 5% among 0.1 min and kept for 2.9 min. For the two ESI modes, the injected volume was 5 μL, the flow rate was 0.35 mL/min, and the auto-sampler was conditioned at 10°C.

**Q Exactive-Orbitrap HRMS detection**. The Exactive-Orbitrap high-resolution mass spectrometer (HRMS) (Thermo Fisher Scientific, San Jose, USA) was equipped with a heat ESI and operated in the full scan mode. The auxiliary gas was at a flow rate of 10 (arbitrary units). The temperatures of capillary and probe heater were kept at 320°C and 300°C, respectively. In ESI^+^ and ESI^–^, the spray voltage was set at 3.5 kV and 2.8 kV, the sheath gas flow velocity was set at 40 (arbitrary units) and 38 (arbitrary units), respectively. The S-lens RF level was set at 50 V. Nitrogen was used for spray stabilization and used as the damping gas in the C-trap. The analysis was performed in full scan mass mode with positive/negative ion swing, namely, ESI^+^ and ESI^–^ were simultaneously scanned. The full MS scan ranges were set from 80 to 1,200 m/z with a resolution of 70,000. The automatic gain control target (the number of ions to fill C-Trap) was set at 3.0e6 with a maximum injection time of 200 millisec. In MS2 mode, the normalized collision energy was set at 20, 40, and 60 eV, and the automatic gain control target was set at 1.0e5 with a maximum injection time of 50 millisec. Besides, the isolation window was set at 1.0 m/z and the resolution was 17,500. The instrument was calibrated every five days by using the manufacturer’s calibration solutions. For positive calibration, the solution contains caffeine, tetrapeptide MRFA, and a mixture of fluorinated phosphazines ultramark 1621. For negative calibration, the solution contains sodium dodecyl sulfate, taurocholic acid sodium salt hydrate, and N-butylamine.

***2.3 Targeted metabolomics analysis***

The targeted metabolomics analysis was performed in the validation phase using a UHPLC-TSQ Altis triple quadrupole mass spectrometer (UHPLC-TSQ-Altis QQQ MS). QC samples in the low, medium, and high concentrations were used to monitor the stability.

**UHPLC separation**. Chromatographic separation was performed by the Thermo Scientific Vanquish Horizon UHPLC system. The column used was an ACQUITY BEH HILIC column (100 mm × 2.1 mm, 1.7 μm) (Waters, Milford, USA), which was employed with a column temperature of 40°C. Mobile phase A was 0.1% formic acid aqueous solution containing 20 mmol/L ammonium acetate, and mobile phase B was acetonitrile solution containing 0.1% formic acid. The elution time was 12.0 min. The elution gradient started with 95% B and held for 2.0 min, then decreased linearly to 60% B at 4.0 min. After kept for 6.0 min with 60% B, the elution gradient increased to 95% among 0.2 min and held for 1.8 min. The injected volume was 3 μL for all samples with a flow rate of 0.30 mL/min, and the temperature of auto-sampler was 10°C.

**TSQ Altis QQQ MS detection**. Thermo Scientific TSQ Altis QQQ MS was equipped with the Thermo Scientific OptaMax NG source housing and operated in ESI^+^. The monitoring mode was selected reaction monitoring. The flow rates of auxiliary gas and sweep gas were 17.1 (arbitrary units), 0.4 (arbitrary units), respectively. The temperatures of ion transfer tube and vaporizer were kept at 325°C and 320°C. The spray voltage and sheath gas flow velocity were set at 3.5 kV and 20.1 (arbitrary units), respectively.

## 3. Qualitative analysis

The candidate biomarkers screened in the discovery phase were only identified by the MS/MS fragment ions through comparison with the online databases and literature. To improve the accuracy of identifying candidate biomarkers in this study, 16 potential biomarkers (L-glutamine, L-valine, L-leucine, L-lysine, L-proline, L-phenylalanine, L-arginine, L-glutamic acid, L-isoleucine, L-methionine, L-carnitine, acetyl-L-carnitine, LPC (P-16:0), LPC (17:0), LPC (14:0), and propionyl-L-carnitine) were selected for qualitative analysis.

The reference compounds for qualitative analysis as the potential biomarkers are provided in Table S8. The stock solution for acetyl-L-carnitine was prepared with 4% hydrochloric acid. The stock solutions for L-valine, L-leucine, L-isoleucine, L-phenylalanine, L-lysine, L-arginine, L-glutamic acid, L-glutamine, L-methionine, L-proline, LPC (14:0), LPC (P-16:0), LPC (17:0), L-carnitine, and propionyl-L-carnitine were prepared with 50% acetonitrile solution. Finally, the final concentrations for L-valine, L-leucine, L-isoleucine, L-phenylalanine, L-lysine, L-arginine, L-glutamic acid, L-glutamine, L-methionine, L-proline, LPC (14:0), LPC (P-16:0), LPC (17:0), L-carnitine, acetyl-L-carnitine, and propionyl-L-carnitine were all 1.0 μg/mL in the mixed standard working solution.

The pre-prepared QC sample was used for qualitative analysis. Pretreatment of the pre-prepared QC sample and the mixed standard working solution was paralleled with the same treatment method as the non-targeted metabolomics analysis.

By comparing the chromatographic peaks, retention time, and mass spectrum information of L-valine, L-leucine, L-isoleucine, L-phenylalanine, L-lysine, L-arginine, L-glutamic acid, L-glutamine, L-methionine, L-proline, LPC (14:0), LPC (P-16:0), LPC (17:0), L-carnitine, acetyl-L-carnitine, and propionyl-L-carnitine in the QC sample and the mixed standard solution, the qualitative analysis results showed that the selected 16 potential biomarkers matched the reference compounds, which suggested the accuracy of identification of a metabolite was high (Figures S7−S9).

## 4. Validation of biological sample quantitative analysis method

The stock solution for acetyl-L-carnitine-d3 was prepared with 4% hydrochloric acid. The stock solution for LPC (20:0)-d4 was prepared with 50% acetonitrile aqueous solution. The stock solution for 20 AA was prepared with 10% methanol. The final internal standard solutions concentrations of 20 AA, acetyl-L-carnitine-d3, and LPC (20:0)-d4 in acetonitrile methanol solution (3:1, v:v) were 10 μg/mL, 500 ng/mL, and 25 ng/mL respectively. All solutions were stored in amber-colored glass volumetric flasks at 4°C refrigerator. The standard solutions were freshly prepared for each batch by adding phosphate buffered saline (simulated serum).^5,6^ because the targeted potential biomarkers are endogenous compounds, it is difficult to prepare analyte free biological matrix.

The standard solutions of L-glutamine, L-valine, L-leucine, L-lysine, L-proline, L-phenylalanine, L-isoleucine, L-arginine, L-glutamic acid, L-methionine, and L-carnitine were prepared with 10% methanol. The standard solutions of LPC (P-16:0), LPC (17:0), LPC (14:0), and propionyl-L-carnitine were prepared with 50% acetonitrile aqueous solution. The standard solution of acetyl-L-carnitine was prepared with 4% hydrochloric acid. The final concentrations of the standard solutions of L-glutamine, L-valine, L-leucine, L-lysine, L-proline, L-phenylalanine, L-isoleucine, L-arginine, L-glutamic acid, L-methionine, L-carnitine, acetyl-L-carnitine, LPC (P-16:0), LPC (17:0), LPC (14:0), and propionyl-L-carnitine are presented in Table S12. Also, QC samples in the low, medium, and high concentrations (LQC, MQC, and HQC) were prepared for validation of biological sample quantitative analysis method, whose concentrations are provided in Table S12.

The validation of biological sample quantitative analysis method included selectivity (mixed solutions containing taurine, sarcosine, creatine, and lauric acid were prepared for analysis, *n*=6), low limit of quantification, the limit of detection, linearity (evaluated by the regression coefficient (R) of the standard curves), intra- (*n*=6) and inter-day precision (*n*=6×3). The extraction recovery (*n*=6), matrix effect (*n*=6), stability (stored in the refrigerator (4°C) and in the auto-sampler (10°C) for 24 hours, *n*=6), dilution effect (diluted 5-fold, *n*=4), and residual effect were also evaluated.^5,7^

The standard curve of each potential biomarker showed well performance in its linear range. The results of low limit of quantification, the limit of detection, standard curves, extraction recovery, matrix effect, precision and accuracy of intra- and inter-day, stability, dilution effect, and residual effect were in line with requirements (Table S13). The validation of biological sample quantitative analysis method results showed that the established targeted metabonomics analysis method was suitable for the large-scale analysis of serum samples from patients with NGT, IFG, and T2DM (Table S13).

## 5 Statistical analysis

## *5.1 Metabolism analysis*

The data collected were processed using the Compound Discoverer (version 3, Thermo Fisher Scientific). The resulting data matrix included the associated retention time, molecular formula, accurate mass, and chromatographic peak area, which were exported to SIMCA (version 14, Umetrics AB, Umea, Sweden) for multivariate analysis. Principal component analysis and orthogonal partial least squares discriminant analysis with unit variance scaling were performed to identify significant discriminating metabolites for the NGT, IFG, and T2DM groups, and 200 permutation tests were conducted to evaluate the risk of over-fitting the model. The differential metabolites were chosen based on their variable importance in the projection (VIP) between groups.^4,8^ A statistical comparison of the characteristics of the NGT, IFG, and T2DM groups was performed using one-way analysis of variance or Kruskal-Wallis test. A conservative Bonferroni correction was used to account for multiple tests.^9^ *P* < 0.05 was considered to represent statistical significance.

The differential metabolites (VIP >1.0 and *P* < 0.05) between groups were identified by searching the Human Metabolome Database^10^ (<https://hmdb.ca/>) and the MassBank of North America^11^ (<https://mona.fiehnlab.ucdavis.edu/>) to match the MS/MS fragment ions with reference substances. Pathway analysis of differential metabolites was performed using the MetaboAnalyst 4 Web service (<https://www.metaboanalyst.ca/>).^12^ Logistic regression (LR) and receiver operating characteristic (ROC) curve analysis with the area under the curve (AUC) were performed using SPSS (version 26, IBM Inc., Armonk, NY, USA). ­­­

For the targeted metabolomics analysis, TraceFinder (version 4.2, Thermo Fisher Scientific) was used for quantitative data processing. Differences in concentrations were analyzed using the Kruskal-Wallis test and adjusted with Bonferroni correction. Violin plots of the concentrations and ROC curves were drawn using Origin 2019 (Electronic Arts Inc., Redwood City, CA, USA). The risks of the potential biomarkers associated with IFG and T2DM were assessed using odds ratio and 95% confidence interval. The odds ratio values were adjusted for sex, age, and body mass index.

## *5.2 Machine learning analysis*

We used the validation phase dataset including 1,132 samples to establish a model to predict the risk of diabetes. The dataset was segmented as the training set and test set using the 70−30 holdout method. The models were trained with the training set to optimize the parameters. Then, the test set was used to evaluate the performance of the model. To explore the most suitable modelling algorithm to construct an IBP prediction model of IFG and T2DM, we compared the performance of prediction models from three different machine learning methods (eXtreme Gradient Boosting (XGBoost), LR, and support vector machine (SVM)) using the test set. The concentrations of the potential biomarkers were set as covariates and disease status was set as the dependent variable. The model was trained using the training set, and the AUC, accuracy, sensitivity, specificity, and precision were used to evaluate the IBP model with the test set, discovery, and test phases. This statistical analysis was performed using Python 3.6, and the XGBoost algorithm was implemented using the machine learning library “Scikit-learn”.

An improvement of the gradient boosting decision tree, the XGBoost^13^ could be used for both classification and regression problems. The XGBoost was one of the boosting tree algorithms, which was a strong classifier from integrated many weak classifiers together. The idea of the XGBoost algorithm was to continuously add trees and continuously segment features to grow a tree. Every time a tree was added, a new function was learned to fit the residual of the last prediction. When we finished training to obtain k trees, we have to predict the score of the sample. Finally, the score corresponding to each tree was added up as the predictive value of the sample. Moreover, the commonly used LR and SVM were applied to compare machine learning methods. LR was a classic algorithm to evaluate the probability of features associated with a disease, which could be used for dichotomy and multi-classification problems.^14^ The weight of the independent feature and predicted probability of disease was obtained by the LR algorithm. The SVM algorithm classified samples by finding the largest hyperplane that differentiated the margins of the two categories of samples. Including polynomial kernel function, Gaussian kernel function, and linear kernel function in the SVM model, in this study, we mainly used linear kernel function.^15^ The key parameters of the XGBoost, LR, and SVM models are provided in Table S9, Table S10, and Table S11, respectively.

To improve the sensitivity of the IBP prediction model for IFG and T2DM, analysis of variance, mutual information, and Gini impurity were used to rank potential biomarkers by importance.^16,17^ The incremental feature selection strategy was used to determine the optimal feature subset. The aim of the analysis of variance was not only to explore the linear relationship between two groups of data but to evaluate the influence of controllable factors by analyzing and studying the contribution of variations from different sources to the total variation.^18^ Mutual information was a measure of the interdependence of variables.^19^ Gini impurity was used for feature selection of the XGBoost algorithm and measures the uncertainty of the result.^20^ The higher the Gini impurity, the more important the potential biomarker.

The AUC, accuracy, sensitivity, specificity, and precision were used to evaluate the model performance.^21,22^ The four parameters of the confusion matrix, true positive (TP), true negative (TN), false positive (FP), and false negative (FN), were used to determine the predicted results of the classifier. Accuracy was the ratio of correctly predicted samples to the total number of test samples. Sensitivity represented the ratio of positive samples correctly classified as positive to all positive samples. Specificity was defined as the ratio of negative samples correctly classified as negative to all negative samples. Precision represented the ratio of positive samples correctly classified as positive to all samples classified as positive. F1-score was an index used in statistics to weigh the accuracy of the classification model. The concrete formulas of the metrics were as follows:

$Accuracy=\frac{TP+TN}{TP+TN+FP+FN}$ (1)

$\mathrm{Re}call/Sensitivity=\frac{TP}{TP+FN}$ (2)

$Specificity=\frac{TN}{TN+FP}$ (3)

$\Pr ecision=\frac{TP}{TP+FP}$ (4)

$F1-score=\frac{2\times\Pr ecision\times\mathrm{Re}call}{\Pr ecision+\mathrm{Re}call}$ (5)

The ROC curve was used to evaluate the predictive performance of the current method across the entire range of algorithm decision values. The ROC curve was illustrated with the true positive rate (TPR) and false positive rate (FPR), which could reveal the relationship between sensitivity and specificity.

Cross-validation was a commonly used technique for evaluating the results of statistical analysis, which could be used to objectively evaluate the performance of classification models. The three widely used cross-validation methods included the independent data set test, n-fold cross-validation test, and jackknife cross-validation test. In this study, the 5-fold cross-validation test and independent test set were employed to evaluate the performance of different models.^23^

## *5.3 Visualization of IBP*

To visualize the IBP, the concentrations of the potential biomarkers were normalized by the following formula:

$B_{(i)}=\frac{B_{(c)}-B_{(\min)}}{B_{(\max)}-B_{(\min)}}\times100$ (6)

Where, *B*(*_c_*), *B*_(min)_, and *B*_(max)_ were the concentration, minimum concentration, and maximum concentration of the potential biomarker before normalization, respectively; *B*(*_i_*) was the value of the potential biomarker after normalization. Then, the mean and standard deviation were calculated from B_(i)_, which was used to draw in a radar.^24^

## 6. Discussion

It is important to identify useful biomarkers for risk assessment of pre-diabetes and T2DM. Therefore, we performed an analysis of serum samples of 1,705 individuals at five clinical centers in China. The study involved discovery, test, and validation phases that used a combination of non-targeted and targeted metabolomics, which presented a rigorous design for the selection of disease biomarkers (Figures 1A−1B). In this way, we created a validated IBP that consisted of LPC (P-16:0), L-isoleucine, L-arginine, L-carnitine, L-phenylalanine, L-glutamic acid, L-lysine, L-methionine, L-leucine, and acetyl-L-carnitine. This was the first time such an IBP had been created, and we also validated its ability to predict IFG and T2DM (Figure S6). Moreover, we established the service website for the public that it could be applied to predict the probability of IFG or T2DM using the IBP, which further implied its potential clinical application. An alternative and more impactful result might have leveraged the IBP to identify, validate, and explore the mechanistic significance of novel metabolites and/or to reveal novel biology.

It is generally believed that the main mechanism of diabetes are insulin resistance and dysfunction of pancreatic islet β-cells.^25^ Insulin resistance refers to the decline in the efficiency of insulin-mediated glucose uptake and utilization, resulting in an increase in blood glucose. Glucose tolerance could be maintained when β-cell function is normal.^26^ It could lead to IFG or at the extreme, T2DM when β-cell dysfunction is present. Insulin resistance and dysfunction of pancreatic islet β-cells caused metabolic disorder of tissue or/and cell, leading to the existence of differential metabolites. Therefore, we designed the detection terminal differential metabolites to predict IFG and T2DM.

There were similarities and differences between our study and other studies, such as the studies from Wang-Sattler et al and Menni et al.^27,28^ Although the three studies all focused on the metabolic differences between patients with pre-diabetes and T2DM, the methods and results of our article were different from the studies of Wang-Sattler et al and Menni et al. First, the studies from Wang-Sattler et al and Menni et al were based on non-targeted metabolomics. Our study used a combination of non-targeted and targeted metabolomics. Second, the differential metabolites identified by the three studies were not all the same. The differential metabolites of most interest to Wang-Sattler et al were glycine, LPC (18:2) and acetylcarnitine; to Menni et al was 3-methyl-2-oxovalerate; to our article were LPC (P-16:0), L-isoleucine, L-arginine, L-carnitine, L-phenylalanine, L-glutamic acid, L-lysine, L-methionine, L-leucine, and acetyl-L-carnitine. Third, in addition to conventional metabolomics analysis methods, we also used machine learning methods to establish prediction models for constructing the IBP of IFG and T2DM, which had the ability to comprehensively and holistically evaluate different biomarkers to predict the risk of diabetes. Furthermore, we established a website of the IBP of IFG and T2DM (<http://pdm.lin-group.cn/>) that can be used by members of the public.

We constructed the IBPs for IFG and T2DM that could objectively reflect the metabolite profile of the disease, which could avoid the use of multiple biomarkers that could confuse the interpretation of the results and avoid the judgment of the overall diagnosis affected by the bias of single or isolated biomarker. The use of the IBPs of IFG and T2DM constructed for diagnosis or treatment of patients with diabetes is convenient. With the quantification of the 10 biomarkers, the subject could be predicted using the established XGBoost model or the established website (<http://pdm.lin-group.cn/>). After an individual diagnosed as IFG using the IBP, interventions can be implemented to reverse or delay the progression of IFG and reduce the incidence of T2DM. Furthermore, for patients diagnosed as T2DM, measures can be taken to prevent its progression and the development of diabetic complications. In addition, for diabetes patients, the efficacy or drug treatment trend could be reflected by the IBP response after the administration of therapeutic drugs.

In conclusion, the present study has provided a strategy for predicting IFG and T2DM using the IBPs by a three-step analysis strategy of discovery, test, and validation. For the first time, to our knowledge, we have not only constructed IBPs that feature a network of biomarkers that are related to IFG and T2DM, but also established a shared service website of the IBP (<http://pdm.lin-group.cn/>) to the public. The IBP could reflect the comprehensive efficiency of integrated biomarkers related to IFG and T2DM, and has the potential risk prediction ability.

## Supplementary References

1. *Definition and diagnosis of diabetes mellitus and intermediate hyperglycemia: report of a WHO/IDF consultation.* Geneva, Switzerland: World Health Organization; 2006.

2. Expert Panel on Detection Evaluation and Treatment of High Blood Cholesterol in Adults. Executive summary of the third report of the National Cholesterol Education Program (NCEP) expert panel on detection, evaluation, and treatment of high blood cholesterol in adults (Adult Treatment Panel III). *JAMA.* 2001;285(19):2486-2497.

3. Yin P, Peter A, Franken H, et al. Preanalytical aspects and sample quality assessment in metabolomics studies of human blood. *Clin Chem.* 2013;59(5):833-845.

4. Luo P, Yin P, Hua R, et al. A Large-scale, multicenter serum metabolite biomarker identification study for the early detection of hepatocellular carcinoma. *Hepatology.* 2018;67(2):662-675.

5. Sugimoto H, Kakehi M, Jinno F. Bioanalytical method for the simultaneous determination of D- and L-serine in human plasma by LC/MS/MS. *Anal Biochem.* 2015;487:38-44.

6. Furtado DZS, de Moura Leite FBV, Barreto CN, et al. Profiles of amino acids and biogenic amines in the plasma of Cri-du-Chat patients. *J Pharm Biomed Anal.* 2017;140:137-145.

7. Olesti E, Rodríguez-Morató J, Gomez-Gomez A, et al. Quantification of endogenous neurotransmitters and related compounds by liquid chromatography coupled to tandem mass spectrometry. *Talanta.* 2019;192:93-102.

8. Trygg J, Holmes E, Lundstedt T. Chemometrics in metabonomics. *J Proteome Res.* 2007;6(2):469-479.

9. Curtin F, Schulz P. Multiple correlations and Bonferroni's correction. *Biol Psychiatry.* 1998;44(8):775-777.

10. Wishart DS, Knox C, Guo AC, et al. HMDB: a knowledgebase for the human metabolome. *Nucleic Acids Res.* 2009;37(Database issue):D603-610.

11. Horai H, Arita M, Kanaya S, et al. MassBank: a public repository for sharing mass spectral data for life sciences. *J Mass Spectrom.* 2010;45(7):703-714.

12. Chong J, Soufan O, Li C, et al. MetaboAnalyst 4.0: towards more transparent and integrative metabolomics analysis. *Nucleic Acids Res.* 2018;46(W1):W486-W494.

13. Chen T, Guestrin C. XGBoost: A scalable tree boosting system. *Proceedings of the 22nd ACM SIGKDD International Conference on Knowledge Discovery and Data Mining*. 2016; Halex, Canada.

14. Pearce J, Ferrier S. Evaluating the predictive performance of habitat models developed using logistic regression. *Ecol Model.* 2000;133(3):225-245.

15. Wang A, Zhao Y, Hou Y, Li Y. A novel construction of SVM compound kernel function. *International Conference on Logistics Systems and Intelligent Management*. 2010; Harbin, Heilongjiang.

16. Nguyen T, Khosravi A, Creighton D, Nahavandi S. Fuzzy system with tabu search learning for classification of motor imagery data. *Biomed Signal Proces.* 2015;20:61-70.

17. Dixon P, Weiner J, Mitchell-Olds T, Woodley R. Bootstrapping the Gini coefficient of inequality. *Ecology.* 1987;68(5):1548-1551.

18. Kerr MK, Martin M, Churchill GA. Analysis of variance for gene expression microarray data. *J Comput Biol.* 2000;7(6):819-837.

19. Chille S, Balocchi R, Garbo AD, et al. Discriminating preictal from interictal states by using coherence measures. *Proceedings of the 25th Annual International Conference of the IEEE Engineering in Medicine and Biology Society*. 2003; Cancun, Mexico.

20. D’Ambrosio A, Tutore VA. Conditional classification trees by weighting the Gini impurity measure. *New Perspectives in Statistical Modeling and Data Analysis*. 2011; Berlin, Heidelberg.

21. Yan L, Zhang H, Goncalves J, et al. An interpretable mortality prediction model for COVID-19 patients. *Nature Machine Intelligence.* 2020;2:283-288.

22. Shu T, Ning W, Wu D, et al. Plasma proteomics identify biomarkers and pathogenesis of COVID-19. *Immunity.* 2020;53:1-15.

23. Xu J, He Y, Qiang B, et al. A novel method for high accuracy sumoylation site prediction from protein sequences. *BMC Bioinformatics.* 2008;9:8.

24. Marins AT, Rodrigues CCR, de Menezes CC, et al. Integrated biomarkers response confirm the antioxidant role of diphenyl diselenide against atrazine. *Ecotoxicol Environ Saf.* 2018;151:191-198.

25. Kahn SE, Hull RL, Utzschneider KM. Mechanisms linking obesity to insulin resistance and type 2 diabetes. *Nature.* 2006;444(7121):840-846.

26. Kahn SE. The importance of β-cell failure in the development and progression of type 2 diabetes. *J Clin Endocr Metab.* 2001;86(9):4047-4058.

27. Wang-Sattler R, Yu Z, Herder C, et al. Novel biomarkers for pre-diabetes identified by metabolomics. *Mol Syst Biol.* 2012;8:615.

28. Menni C, Fauman E, Erte I, et al. Biomarkers for type 2 diabetes and impaired fasting glucose using a nontargeted metabolomics approach. *Diabetes.* 2013;62(12):4270-4276.

## Supplementary Figures


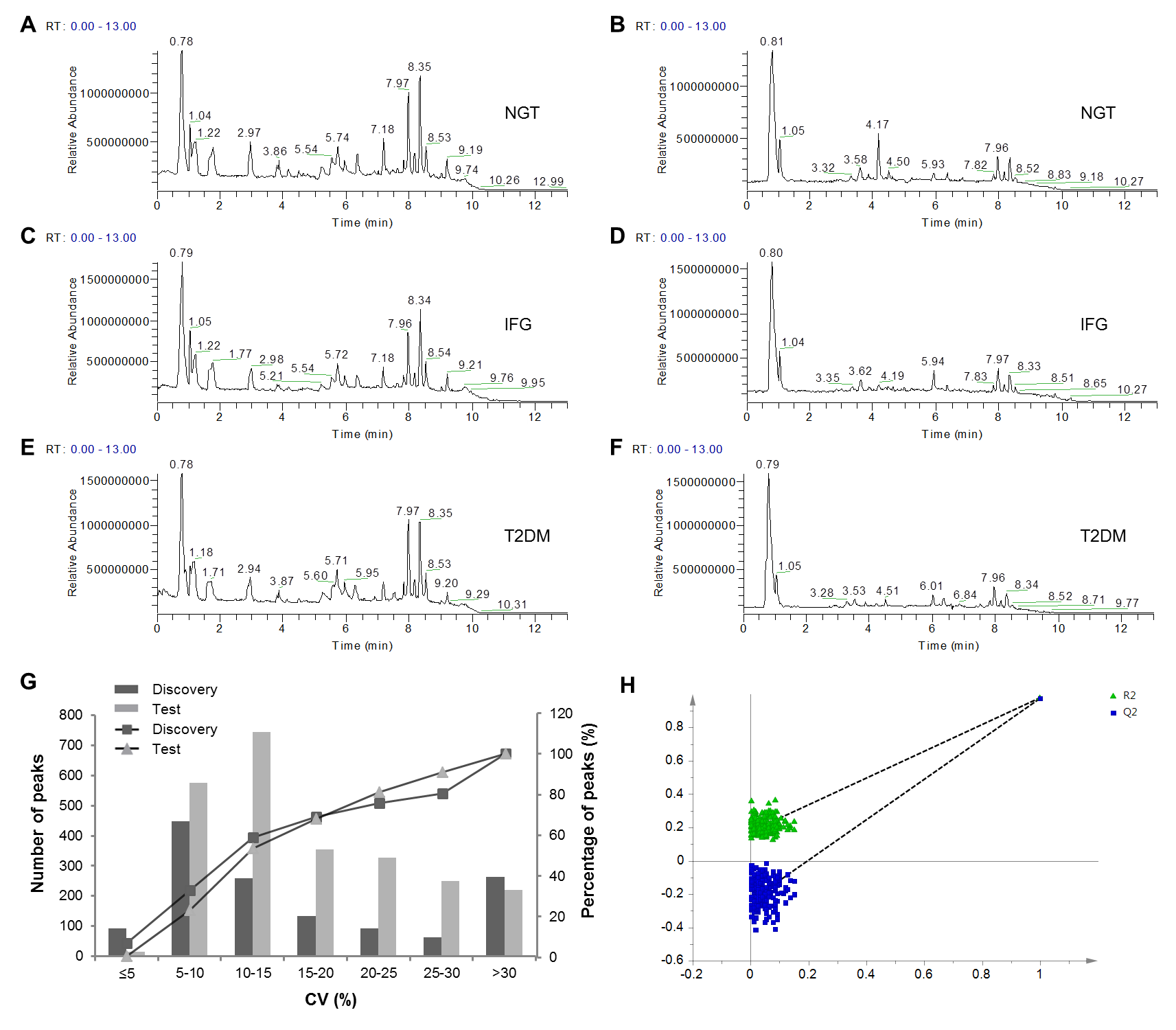


**Figure S1.** **Metabonomics characteristic information**. TIC was collected in ESI^+^ (A) and ESI^–^ (B) in the NGT group. TIC was collected in ESI^+^ (C) and ESI^–^ (D) in the IFG group. TIC was collected in ESI^+^ (E) and ESI^–^ (F) in the T2DM group. (G) CV distribution of the peaks in a combinational dataset of positive and negative electrospray ionization modes from the discovery and test sets, respectively. The columns and lines represent the number of peaks and accumulative percentage of peaks in the corresponding CV interval, respectively. After removing the missing values using the 80% rule, 60% and 94% peaks had CV below 15% and 30%, respectively. (H) Cross-validation plot with a permutation test repeated 200 times in positive electrospray ionization mode. The intercepts of R2=(0.0, 0.175) and Q2=(0.0, –0.24) illustrated the orthogonal partial least squares discriminant analysis model was not over-fitted. Abbreviations: CV, coefficients of variation; TIC, total ion chromatogram; ESI, electrospray ionization; NGT, normal glucose tolerance; IFG, impaired fasting glucose; T2DM, type 2 diabetes mellitus.


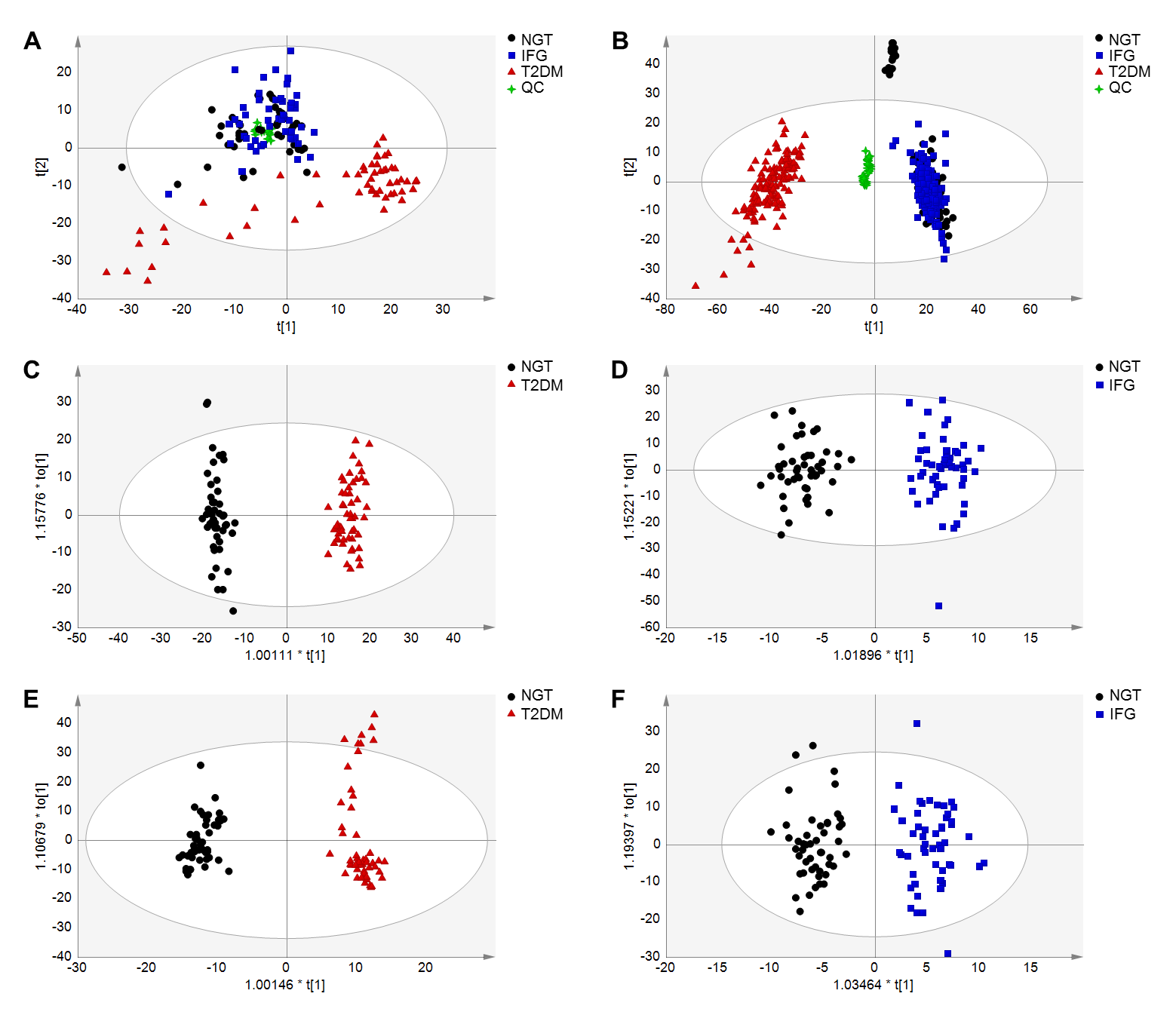


**Figure S2. Metabolomic characterization**. (A) PCA score plot of ESI^−^ in the discovery phase. QC samples clustered together. NGT, *N=*48; IFG, *N=*51; T2DM, *N=*54. (B) PCA score plot of ESI^+^ in the test phase. The QC samples clustered together. NGT, *N=*135; IFG, *N=*147; T2DM, *N=*138. (C) OPLS-DA score plot of T2DM *vs.* NGT from ESI^+^ in the discovery phase. (D) OPLS-DA score plot of IFG *vs.* NGT from ESI^+^ in the discovery phase. (E) OPLS-DA score plot of T2DM *vs.* NGT from ESI^−^ in the discovery phase. (F) OPLS-DA score plot of IFG *vs.* NGT from ESI^−^ in the discovery phase. Abbreviations: NGT, normal glucose tolerance; IFG, impaired fasting glucose; T2DM, type 2 diabetes mellitus; QC, quality control; ESI, electrospray ionization; PCA, principal component analysis; OPLS-DA, orthogonal partial least squares discriminant analysis.


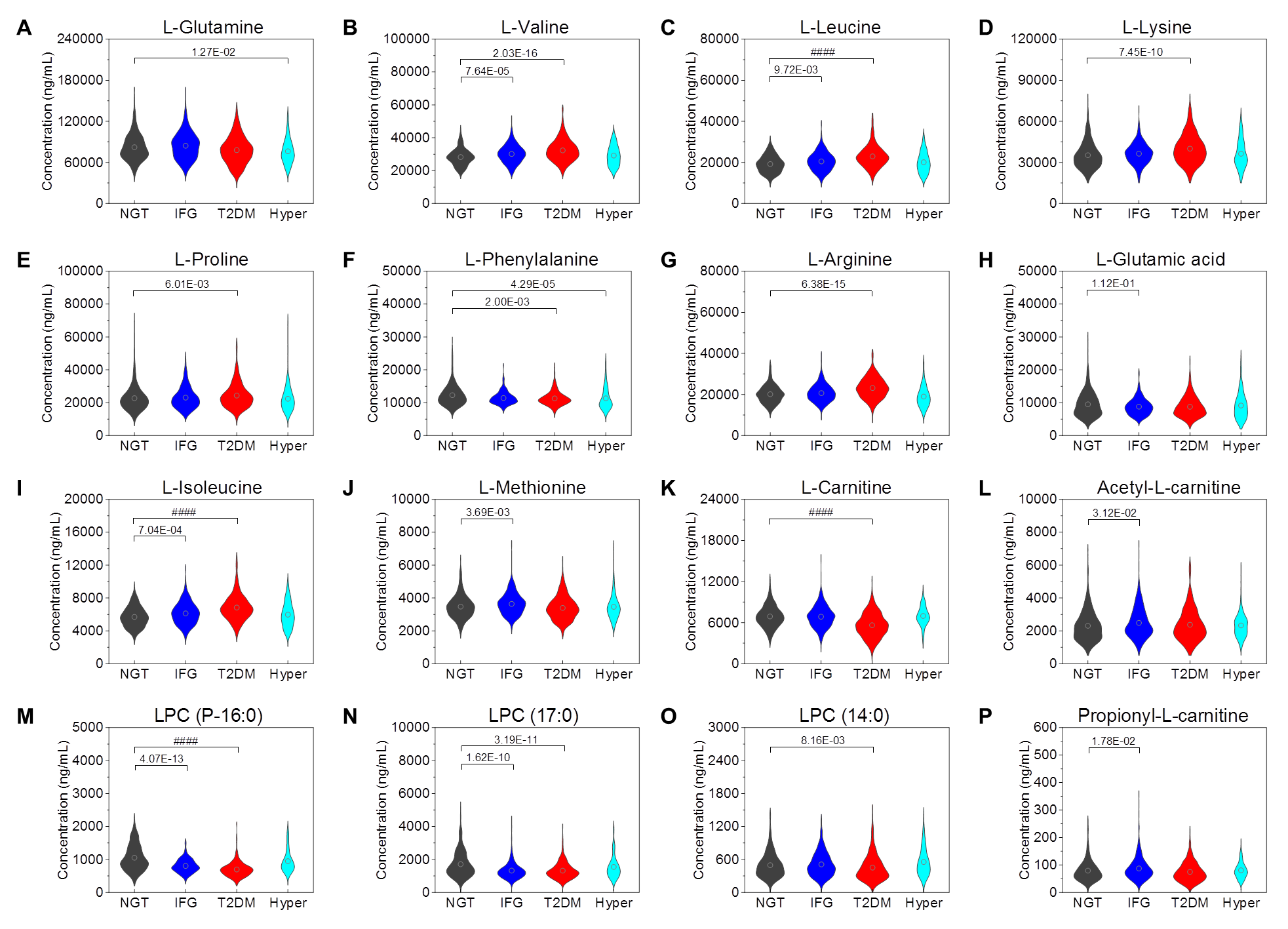


**Figure S3. Differences in concentrations of the potential biomarkers in serum of the groups**. Differences in concentrations in serum of (A) L-glutamine, (B) L-valine, (C) L-leucine, (D) L-lysine, (E) L-proline, (F) L-phenylalanine, (G) L-arginine, (H) L-glutamic acid, (I) L-isoleucine, (J) L-methionine, (K) L-carnitine, (L) acetyl-L-carnitine, (M) LPC (P-16:0), (N) LPC (17:0), (O) LPC (14:0), and (P) propionyl-L-carnitine. In figure, NGT, *N=*312; IFG, *N=*311; T2DM, *N=*363; Hyper, *N=*146. Group differences in concentrations were analyzed using the Kruskal-Wallis test. The significance values were adjusted using the Bonferroni correction for multiple tests, and #### represents an adjusted *P* < 1.00×10^-17^. Abbreviations: NGT, normal glucose tolerance; IFG, impaired fasting glucose; T2DM, type 2 diabetes mellitus; Hyper, hyperlipidemia; LPC, lysophosphatidylcholine.


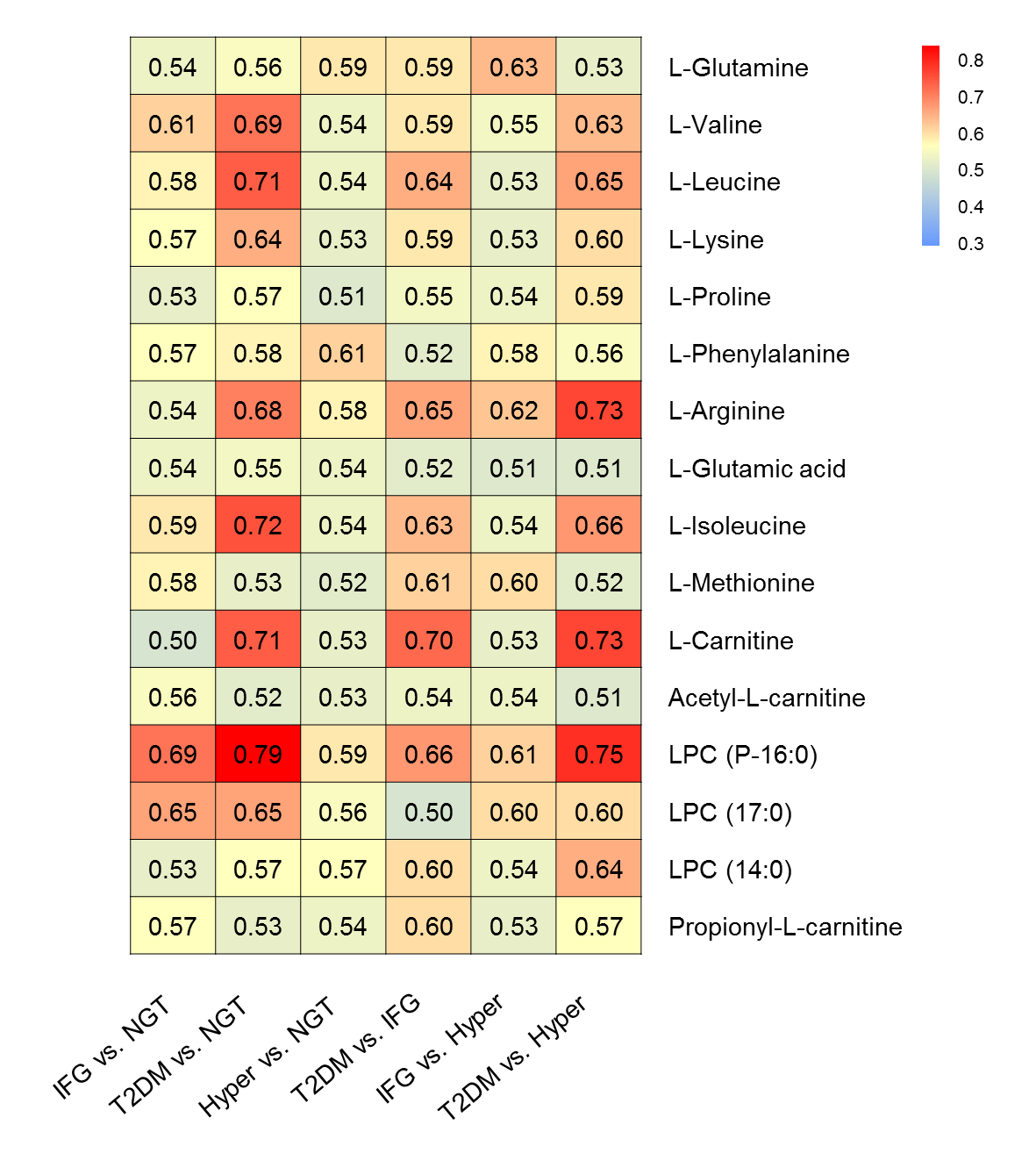


**Figure S4. Classifier performance for diabetes discrimination using 16 potential biomarkers.** Data are the areas under the receiver operating characteristic curves. In figure, NGT, *n=*312; IFG, *n=*311; T2DM, *n=*363; Hyper, *n=*146. Abbreviations: NGT, normal glucose tolerance; IFG, impaired fasting glucose; T2DM, type 2 diabetes mellitus; Hyper, hyperlipidemia; LPC, lysophosphatidylcholine.


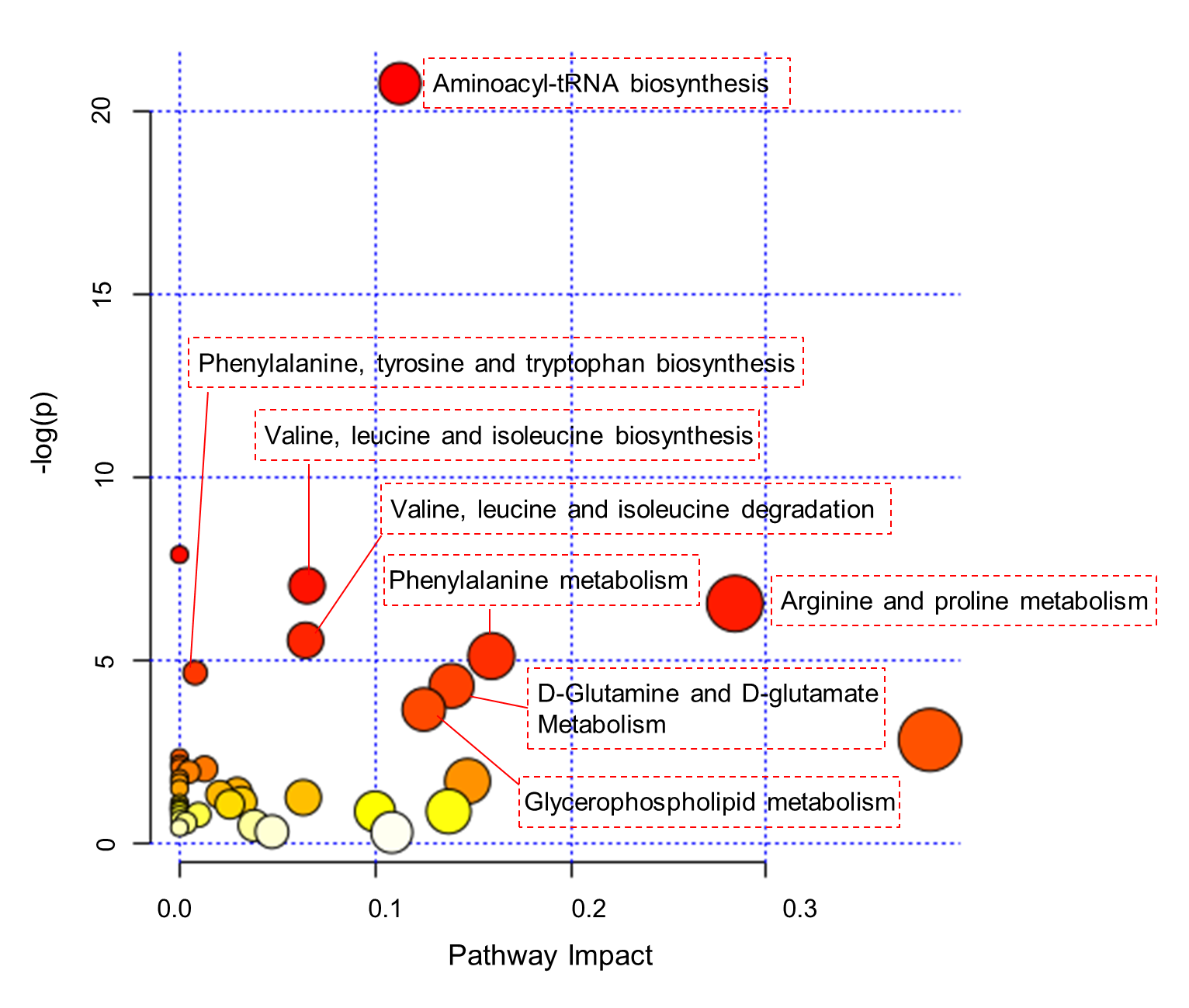


**Figure S5. The pathway analysis of the biomarker candidates.** Metabolite pathways related to the perturbations of diabetes are performed by the website of MetaboAnalyst based on all the differential metabolites listed in Tables S1-S2.


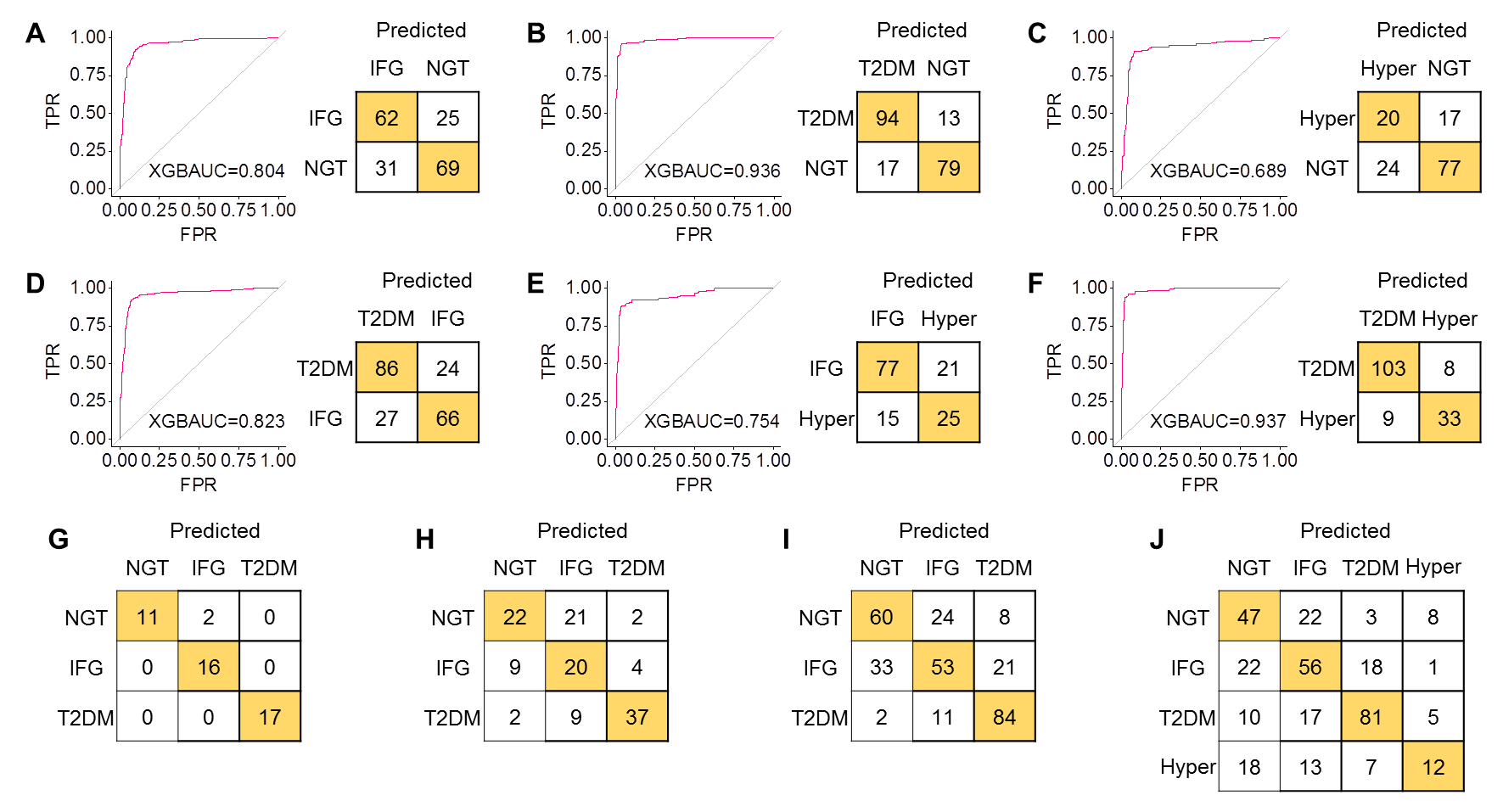


**Figure S6. Diagnostic performance of the integrated biomarker profiling**. (A−F) Integrated biomarker profiling performance based on the XGBoost model, for the comparisons of two groups: IFG *vs.* NGT (A), T2DM *vs.* NGT (B), Hyper *vs.* NGT (C), T2DM *vs.* IFG (D), IFG *vs.* Hyper (E), and T2DM *vs.* Hyper (F); among three groups during the discovery phase (G) and test phase (H), and in the test set (I); among four groups in the test set (J) (shown above as confusion matrices). Abbreviations: NGT, normal glucose tolerance; IFG, impaired fasting glucose; T2DM, type 2 diabetes mellitus; Hyper, hyperlipidemia.


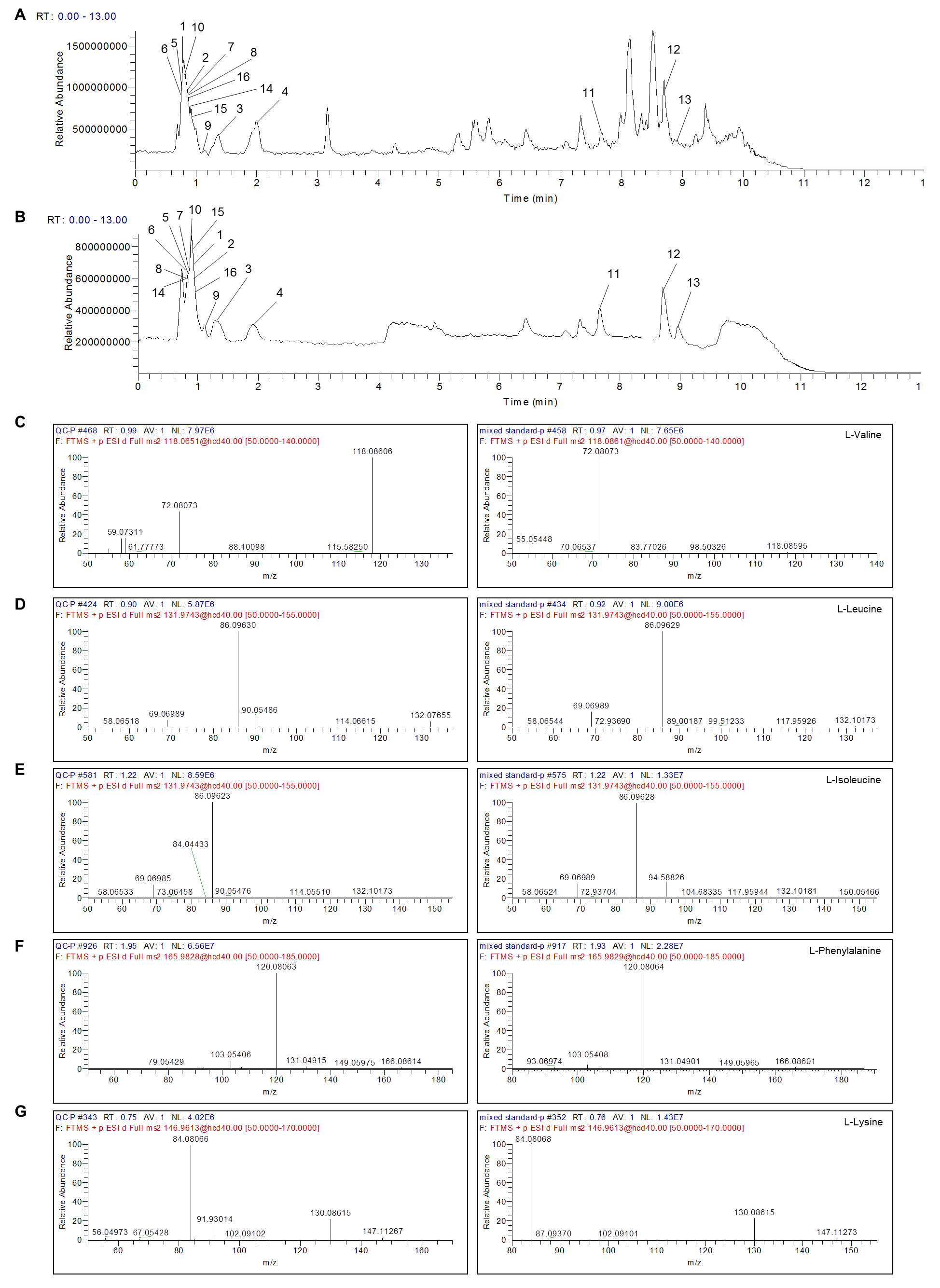


**Figure S7. Qualitative analysis result**. Total ion chromatogram of quality control sample (A) and mixed standard (B) in positive ion mode. In figure, the numbers 1-16 represent L-valine, L-leucine, L-isoleucine, L-phenylalanine, L-lysine, L-arginine, L-glutamic acid, L-glutamine, L-methionine, L-proline, LPC (14:0), LPC (P-16:0), LPC (17:0), L-carnitine, acetyl-L-carnitine, and propionyl-L-carnitine, respectively. Mass spectrum of L-valine (C), L-leucine (D), L-isoleucine (E), L-phenylalanine (F), and L-lysine (G) in the quality control sample (left) and mixed standard (right). Abbreviation: LPC, lysophosphatidylcholine.


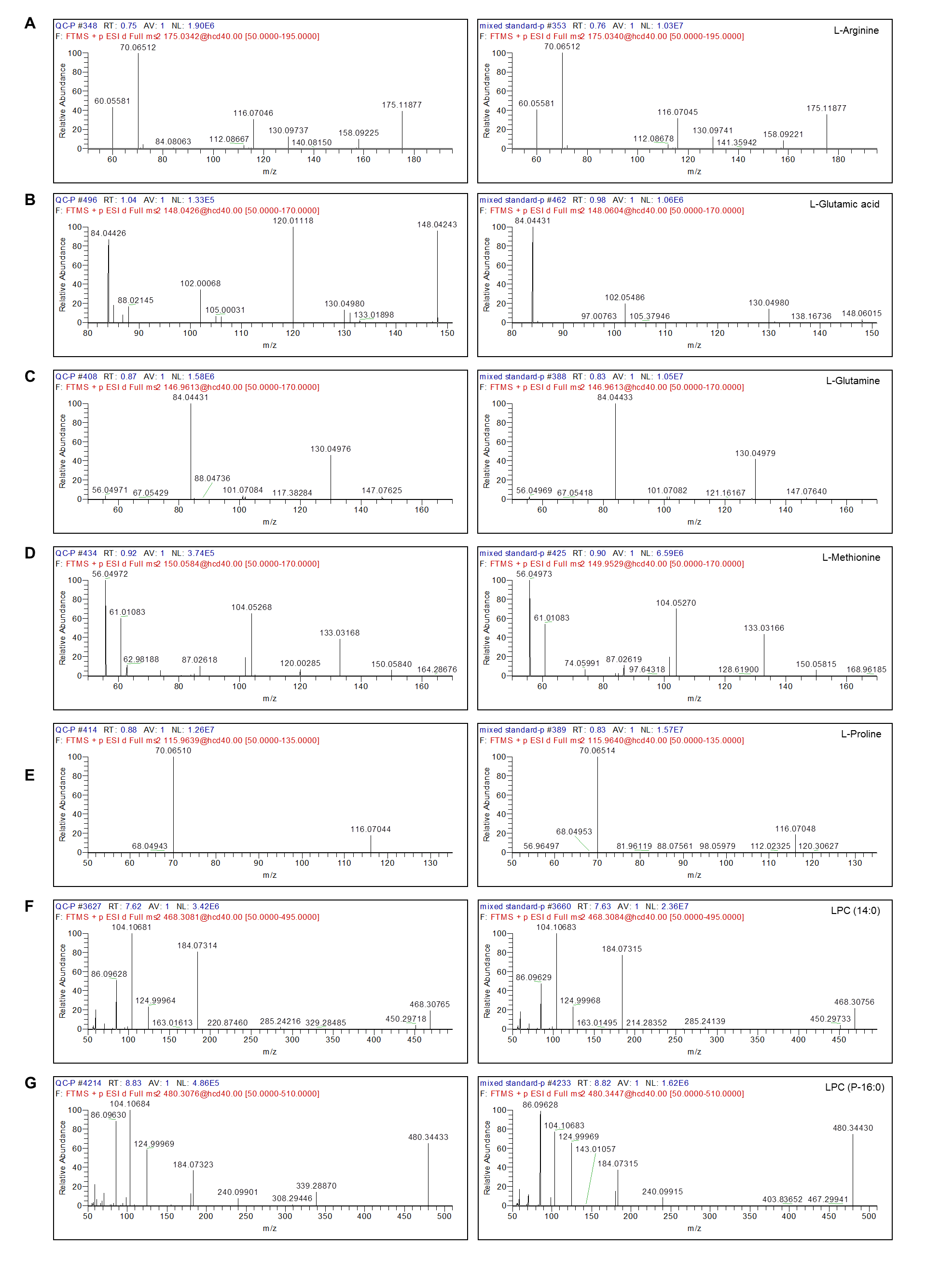


**Figure S8. Qualitative analysis mass spectrogram**. Mass spectrogram of L-arginine (A), L-glutamic acid (B), L-glutamine (C), L-methionine (D), L-proline (E), LPC (14:0) (F), and LPC (P-16:0) (G) in the quality control sample (left) and mixed standard (right). Abbreviation: LPC, lysophosphatidylcholine.


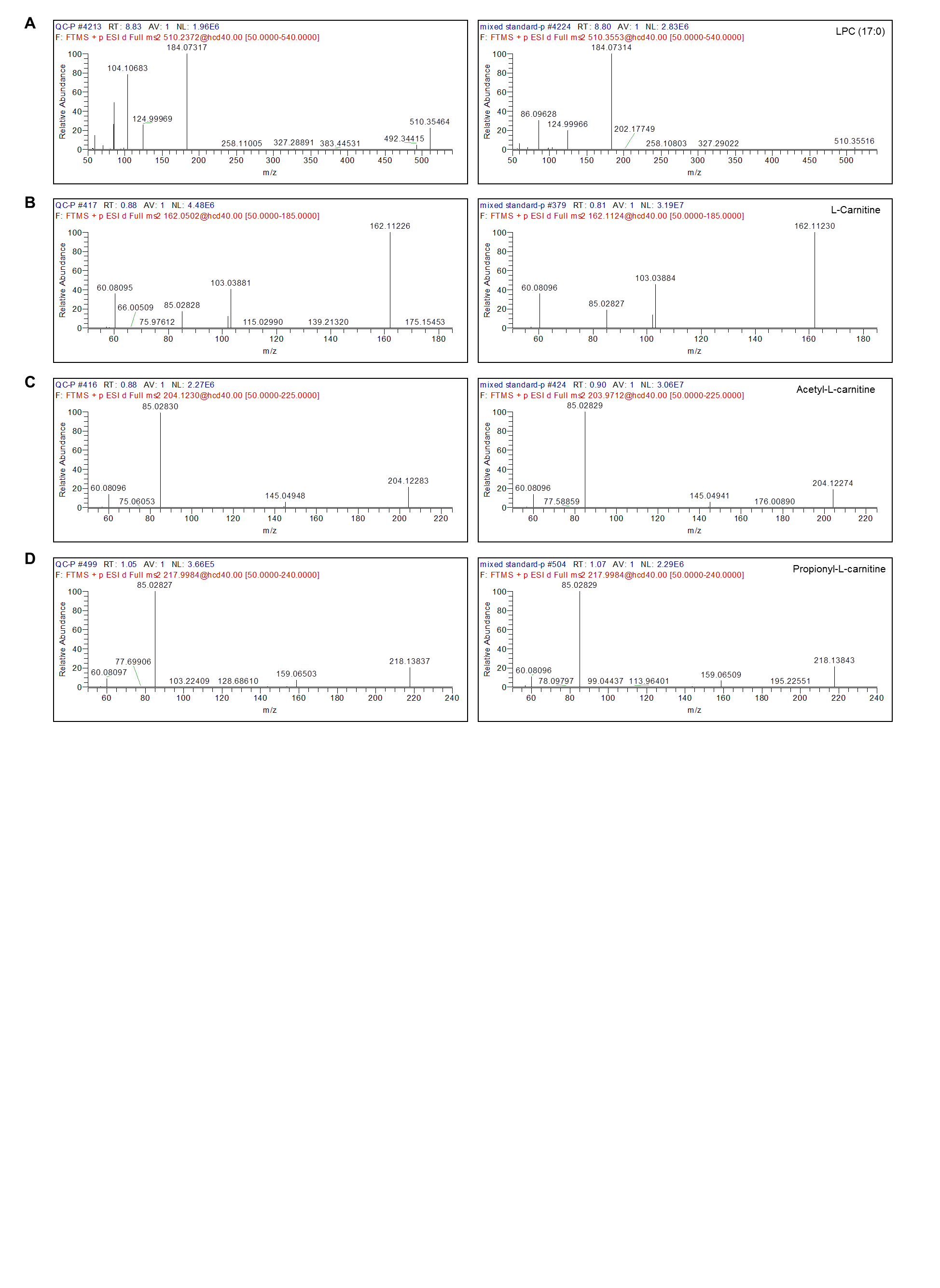


**Figure S9. Qualitative analysis mass spectrogram**. Mass spectrogram of LPC (17:0) (A), L-carnitine (B), acetyl-L-carnitine (C), and propionyl-L-carnitine (D) in the quality control sample (left) and mixed standard (right).

## Supplementary Tables

**Table S1. Biomarker candidates in serum of IFG individuals.**

| **No.** | **ESI mode** | **RT [min]** | **m/z** | **Formula** | **BMDB ID** | **Metabolites** | **VIP** | **Fold Change** | ***P* value** |
| --- | --- | --- | --- | --- | --- | --- | --- | --- | --- |
| 1 | – | 0.842 | 134.02127 | C_4_H_6_O_5_ | HMDB0000744 | Malic acid | 1.38 | 1.18 | 5.68E-03 |
| 2 | – | 0.849 | 147.05286 | C_5_H_9_NO_4_ | HMDB0000148 | Glutamic acid | 2.06 | 1.16 | 1.66E-03 |
| 3 | – | 0.853 | 168.02799 | C_5_H_4_N_4_O_3_ | HMDB0000289 | Uric acid^#^ | 1.05 | 1.11 | 3.59E-02 |
| 4 | + | 0.872 | 161.10491 | C_7_H_15_NO_3_ | HMDB0000062 | L-Carnitine | 3.06 | 1.18 | 8.35E-06 |
| 5 | + | 0.876 | 129.07876 | C_6_H_11_NO_2_ | HMDB0000070 | Pipecolic acid | 3.11 | 1.17 | 2.24E-06 |
| 6 | + | 0.877 | 115.06307 | C_5_H_9_NO_2_ | HMDB0000162 | L-Proline | 1.30 | 1.14 | 2.52E-02 |
| 7 | + | 0.885 | 203.11542 | C_9_H_17_NO_4_ | HMDB0000201 | Acetyl-L-carnitine | 1.74 | 1.15 | 7.58E-03 |
| 8 | + | 0.890 | 217.13097 | C_10_H_19_NO_4_ | HMDB0000824 | Propionylcarnitine | 1.45 | 1.19 | 7.14E-03 |
| 9 | – | 0.904 | 90.03143 | C_3_H_6_O_3_ | HMDB0000190 | Lactic acid | 2.01 | 1.12 | 1.33E-03 |
| 10 | + | 0.906 | 149.05082 | C_5_H_11_NO_2_S | HMDB0000696 | Methionine | 5.32 | 0.21 | 6.29E-26 |
| 11 | + | 0.910 | 147.03526 | C_5_H_9_NO_2_S | HMDB0059611 | Thiomorpholine 3-carboxylate | 3.99 | 0.37 | 1.03E-17 |
| 12 | + | 1.055 | 146.06897 | C_5_H_10_N_2_O_3_ | HMDB0000641 | Glutamine | 3.34 | 1.37 | 1.01E-05 |
| 13 | + | 1.119 | 168.0281 | C_5_H_4_N_4_O_3_ | HMDB0000289 | Uric acid | 1.51 | 1.13 | 1.70E-02 |
| 14 | + | 1.119 | 203.11541 | C_9_H_17_NO_4_ | HMDB0000201 | Acetyl-L-carnitine^#^ | 1.67 | 1.15 | 8.38E-03 |
| 15 | + | 1.128 | 164.04712 | C_9_H_8_O_3_ | HMDB0001713 | m-Coumaric acid | 1.38 | 1.09 | 3.68E-02 |
| 16 | + | 1.135 | 181.07362 | C_9_H_11_NO_3_ | HMDB0000158 | Tyrosine | 1.39 | 1.09 | 3.54E-02 |
| 17 | – | 1.238 | 104.0471 | C_4_H_8_O_3_ | HMDB0000357 | 3-Hydroxybutyric acid | 3.97 | 3.89 | 1.53E-14 |
| 18 | + | 1.256 | 115.06314 | C_5_H_9_NO_2_ | HMDB0003411 | D-Proline | 2.76 | 1.16 | 7.22E-05 |
| 19 | + | 1.362 | 217.13095 | C_10_H_19_NO_4_ | HMDB0062514 | O-Propanoyl carnitine | 1.64 | 1.26 | 4.00E-03 |
| 20 | + | 1.774 | 165.07877 | C_9_H_11_NO_2_ | METPA0264 | D-Phenylalanine | 1.83 | 0.72 | 3.23E-02 |
| 21 | – | 3.386 | 130.06273 | C_6_H_10_O_3_ | HMDB0000491 | 3-Methyl-2-oxovaleric acid | 1.61 | 1.18 | 8.85E-03 |
| 22 | – | 3.639 | 130.06273 | C_6_H_10_O_3_ | HMDB0000695 | 4-Methyl-2-oxovaleric acid | 1.81 | 1.21 | 2.28E-02 |
| 23 | + | 4.080 | 210.1366 | C_11_H_18_N_2_O_2_ | HMDB0034276 | Cyclo (leu-pro)^#^ | 2.21 | 1.74 | 1.45E-05 |
| 24 | + | 4.352 | 351.15787 | C_20_H_21_N_3_O_3_ | HMDB0029090 | TRP-Phe | 1.98 | 1.27 | 1.83E-03 |
| 25 | + | 7.568 | 467.30047 | C_22_H_46_NO_7_P | HMDB0010379 | LPC (14:0) | 3.02 | 1.41 | 1.62E-05 |
| 26 | + | 7.706 | 423.33396 | C_25_H_45_NO_4_ | HMDB0006469 | Linoleyl carnitine | 1.64 | 0.89 | 4.17E-02 |
| 27 | + | 8.205 | 507.33179 | C_25_H_50_NO_7_P | HMDB0011512 | LPE (20:1) | 1.47 | 1.15 | 3.58E-02 |
| 28 | + | 8.347 | 545.34767 | C_28_H_52_NO_7_P | HMDB0010393 | LPC (20:3) | 1.24 | 1.14 | 3.56E-02 |
| 29 | + | 8.643 | 479.33665 | C_24_H_50_NO_6_P | HMDB0010407 | LPC (P-16:0) | 1.76 | 0.90 | 1.35E-02 |
| 30 | + | 8.825 | 507.36824 | C_26_H_54_NO_6_P | HMDB0013122 | LPC (P-18:0) | 1.76 | 0.90 | 2.25E-02 |
| 31 | + | 9.823 | 281.27113 | C_18_H_35_NO | HMDB0002117 | Oleamide | 1.19 | 0.75 | 3.99E-02 |

Abbreviations: ESI, electrospray ionization; RT, retention time; VIP, variable importance in the projection; TRP-Phe, tryptophyl-phenylalanine; LPC, lysophosphatidylcholine; LPE, lysophosphatidylethanolamine. “+” and “–” represent positive and negative ion modes. ^#^Metabolite may be an isomer.

**Table S2. Biomarker candidates in serum of T2DM patients.**

| **No.** | **ESI mode** | **RT [min]** | **m/z** | **Formula** | **BMDB ID** | **Metabolites** | **VIP** | **Fold Change** | ***P* value** |
| --- | --- | --- | --- | --- | --- | --- | --- | --- | --- |
| 1 | + | 0.793 | 174.11073 | C_6_H_14_N_4_O_2_ | HMDB0000517 | Arginine | 1.07 | 0.67 | 1.13E-06 |
| 2 | – | 0.849 | 147.05286 | C_5_H_9_NO_4_ | HMDB0000148 | Glutamic acid | 1.19 | 1.22 | 6.13E-03 |
| 3 | + | 0.866 | 113.05873 | C_4_H_7_N_3_O | HMDB0000562 | Creatinine | 1.12 | 1.19 | 2.82E-04 |
| 4 | + | 0.876 | 129.07876 | C_6_H_11_NO_2_ | HMDB0000070 | Pipecolic acid | 1.26 | 1.20 | 7.40E-06 |
| 5 | + | 0.885 | 203.11542 | C_9_H_17_NO_4_ | HMDB0000201 | Acetyl-L-carnitine | 1.22 | 1.32 | 3.46E-06 |
| 6 | + | 0.888 | 117.07869 | C_5_H_11_NO_2_ | HMDB0000883 | Valine | 4.21 | 1.19 | 4.30E-03 |
| 7 | – | 0.904 | 90.03143 | C_3_H_6_O_3_ | HMDB0000190 | Lactic acid | 2.10 | 1.52 | 1.13E-15 |
| 8 | + | 0.906 | 149.05082 | C_5_H_11_NO_2_S | HMDB0000696 | Methionine | 2.48 | 0.16 | 1.35E-31 |
| 9 | + | 0.910 | 147.03526 | C_5_H_9_NO_2_S | HMDB0059611 | Thiomorpholine 3-carboxylate | 2.30 | 0.24 | 9.36E-34 |
| 10 | + | 0.911 | 131.09438 | C_6_H_13_NO_2_ | HMDB0000687 | Leucine | 2.10 | 0.61 | 1.19E-19 |
| 11 | + | 0.932 | 146.1055 | C_6_H_14_N_2_O_2_ | HMDB0000182 | Lysine | 1.02 | 1.26 | 2.55E-02 |
| 12 | + | 1.055 | 146.06897 | C_5_H_10_N_2_O_3_ | HMDB0000641 | Glutamine | 1.02 | 1.25 | 9.14E-04 |
| 13 | – | 1.081 | 244.06907 | C_9_H_12_N_2_O_6_ | HMDB0000296 | Uridine | 1.29 | 0.77 | 1.19E-05 |
| 14 | – | 1.084 | 129.04233 | C_5_H_7_NO_3_ | HMDB0000267 | Pyroglutamic acid | 1.87 | 0.79 | 1.24E-10 |
| 15 | – | 1.092 | 192.02671 | C_6_H_8_O_7_ | HMDB0000094 | Citric acid | 1.25 | 0.79 | 8.07E-05 |
| 16 | + | 1.256 | 115.06314 | C_5_H_9_NO_2_ | HMDB0003411 | D-Proline | 1.08 | 1.19 | 1.90E-03 |
| 17 | + | 1.280 | 131.09424 | C_6_H_13_NO_2_ | HMDB0000172 | Isoleucine | 2.23 | 0.49 | 1.81E-22 |
| 18 | + | 1.288 | 175.05821 | C_5_H_9_N_3_O_4_ | HMDB0003157 | Guanidinosuccinic acid | 1.98 | 0.68 | 3.07E-14 |
| 19 | – | 1.824 | 116.04705 | C_5_H_8_O_3_ | HMDB0000720 | Levulinic acid | 1.03 | 0.79 | 1.51E-03 |
| 20 | + | 2.005 | 165.07895 | C_9_H_11_NO_2_ | HMDB0000159 | L-Phenylalanine | 1.55 | 0.56 | 1.43E-07 |
| 21 | + | 3.095 | 187.06311 | C_11_H_12_N_2_O_2_ | HMDB0000929 | Tryptophan | 2.27 | 0.19 | 5.42E-20 |
| 22 | + | 3.558 | 210.13665 | C_11_H_18_N_2_O_2_ | HMDB0034276 | Cyclo (leu-pro)^#^ | 1.10 | 0.71 | 3.08E-05 |
| 23 | + | 3.850 | 288.1544 | C_10_H_20_N_6_O_4_ | HMDB0028725 | Asn-Arg | 1.45 | 0.78 | 3.89E-08 |
| 24 | + | 4.240 | 312.14695 | C_18_H_20_N_2_O_3_ | HMDB0013302 | Phe-phe | 1.14 | 1.65 | 1.04E-03 |
| 25 | + | 4.352 | 351.15787 | C_20_H_21_N_3_O_3_ | HMDB0029090 | TRP-Phe | 1.43 | 1.47 | 4.99E-07 |
| 26 | + | 5.804 | 584.26229 | C_33_H_36_N_4_O_6_ | HMDB0000054 | Bilirubin | 1.79 | 0.33 | 1.85E-16 |
| 27 | – | 6.214 | 370.18108 | C_19_H_30_O_5_S | HMDB0002759 | Androsterone sulfate | 1.50 | 0.37 | 1.57E-08 |
| 28 | + | 6.504 | 582.24667 | C_33_H_34_N_4_O_6_ | HMDB0001008 | Biliverdin | 1.06 | 0.70 | 3.95E-03 |
| 29 | + | 7.242 | 182.07287 | C_13_H_10_O | HMDB0013163 | 2-Hydroxyfluorene | 1.46 | 1.30 | 2.78E-09 |
| 30 | – | 7.436 | 379.24841 | C_18_H_38_NO_5_P | HMDB0000277 | Sphingosine 1-phosphate | 1.86 | 0.75 | 4.63E-10 |
| 31 | + | 7.568 | 467.30047 | C_22_H_46_NO_7_P | HMDB0010379 | LPC (14:0) | 1.26 | 1.52 | 1.34E-05 |
| 32 | + | 7.909 | 519.33183 | C_26_H_50_NO_7_P | HMDB0010386 | LPC (18:2) | 1.02 | 0.83 | 6.56E-04 |
| 33 | + | 8.106 | 103.09952 | C_5_H_13_NO | HMDB0000097 | Choline | 2.03 | 1.70 | 8.91E-19 |
| 34 | + | 8.138 | 234.16139 | C_15_H_22_O_2_ | HMDB0030016 | Valerenic acid | 1.58 | 0.68 | 3.10E-10 |
| 35 | – | 8.373 | 481.31658 | C_23_H_48_NO_7_P | HMDB0011129 | LPE (18:0) | 1.34 | 0.85 | 1.43E-06 |
| 36 | + | 8.630 | 437.28961 | C_21_H_44_NO_6_P | HMDB0011152 | PE (P-16:0e/0:0) | 1.17 | 0.71 | 1.42E-07 |
| 37 | + | 8.643 | 479.33665 | C_24_H_50_NO_6_P | HMDB0010407 | LPC (P-16:0) | 1.94 | 0.67 | 2.81E-14 |
| 38 | + | 8.825 | 507.36824 | C_26_H_54_NO_6_P | HMDB0013122 | LPC (P-18:0) | 1.99 | 0.63 | 8.95E-16 |
| 39 | + | 8.861 | 509.34751 | C_25_H_52_NO_7_P | HMDB0012108 | LPC (17:0) | 1.46 | 0.73 | 1.77E-08 |
| 40 | + | 9.392 | 549.37806 | C_28_H_56_NO_7_P | HMDB0011148 | PC (18:1(9Z)e/2:0) | 1.89 | 0.52 | 1.42E-15 |
| 41 | + | 10.339 | 805.55834 | C_46_H_80_NO_8_P | HMDB0008179 | PC (18:3/20:3) | 1.48 | 0.51 | 2.73E-09 |
| 42 | + | 10.662 | 582.24623 | C_33_H_34_N_4_O_6_ | HMDB0001008 | Biliverdin^#^ | 1.77 | 0.24 | 1.22E-19 |

Abbreviations: ESI, electrospray ionization; RT, retention time; VIP, variable importance in the projection; Asn-arg, asparaginyl-arginine; Phe-phe, phenylalanyl-phenylalanine; TRP-Phe, tryptophyl-phenylalanine; LPC, lysophosphatidylcholine; PE, phosphatidylethanolamine; LPE, lysophosphatidylethanolamine; PC, phosphatidylcholine. “+” and “–” represent positive and negative ion modes. ^#^Metabolite may be an isomer.

**Table S3. Results of the logistic regression analysis of the biomarker candidates.**

| **No.** | **Metabolites** | **IFG *vs.* NGT** | | | **T2DM *vs.* NGT** | | |
| --- | --- | --- | --- | --- | --- | --- | --- |
|  |  | **Coefficient** | **S.E.** | ***P* value** | **Coefficient** | **S.E.** | ***P* value** |
| 1 | Arginine | –0.001 | 0.119 | 9.93E-01 | 1.603 | 0.215 | 7.84E-14 |
| 2 | Creatinine | 0.331 | 0.124 | 7.78E-03 | 0.462 | 0.133 | 5.40E-04 |
| 3 | L-Carnitine | 0.275 | 0.123 | 2.55E-02 | 0.079 | 0.122 | 5.16E-01 |
| 4 | L-Proline | –0.053 | 0.120 | 6.59E-01 | 0.299 | 0.129 | 2.07E-02 |
| 5 | Malic acid | –0.014 | 0.119 | 9.04E-01 | 0.600 | 0.145 | 3.47E-05 |
| 6 | Glutamine | –0.240 | 0.121 | 4.77E-02 | –0.477 | 0.131 | 2.56E-04 |
| 7 | Propionyl carnitine | 0.331 | 0.130 | 1.08E-02 | 1.195 | 0.186 | 1.27E-10 |
| 8 | Acetyl-L-carnitine | 0.657 | 0.144 | 4.88E-06 | 1.204 | 0.200 | 1.93E-09 |
| 9 | Glutamic acid | –0.271 | 0.128 | 3.39E-02 | –0.233 | 0.127 | 4.66E-02 |
| 10 | Valine | –0.112 | 0.120 | 3.51E-01 | 1.120 | 0.171 | 5.48E-11 |
| 11 | Uric acid | 0.123 | 0.120 | 3.06E-01 | –0.321 | 0.126 | 1.09E-02 |
| 12 | Leucine | –0.197 | 0.121 | 1.05E-01 | 0.652 | 0.141 | 3.80E-06 |
| 13 | Lactic acid | 0.077 | 0.120 | 5.21E-01 | 0.870 | 0.160 | 5.95E-08 |
| 14 | Lysine | –0.291 | 0.123 | 1.81E-02 | 0.082 | 0.122 | 4.99E-01 |
| 15 | Methionine | 0.193 | 0.121 | 1.11E-01 | 0.207 | 0.124 | 4.38E-02 |
| 16 | Uridine | –0.336 | 0.124 | 6.68E-03 | –0.027 | 0.121 | 8.26E-01 |
| 17 | Uric acid^#^ | 0.215 | 0.123 | 7.97E-02 | –0.420 | 0.129 | 1.09E-03 |
| 18 | Pipecolic acid | –0.283 | 0.127 | 2.56E-02 | –2.529 | 0.322 | 4.10E-15 |
| 19 | Thiomorpholine 3-carboxylate | 0.308 | 0.125 | 1.33E-02 | –2.545 | 0.307 | 1.18E-16 |
| 20 | Pyroglutamic acid | –0.751 | 0.194 | 1.05E-04 | –1.064 | 0.205 | 2.02E-07 |
| 21 | Citric acid | –0.313 | 0.125 | 1.19E-02 | –0.096 | 0.122 | 4.30E-01 |
| 22 | D-Proline | –0.580 | 0.157 | 2.11E-04 | –0.288 | 0.130 | 2.73E-02 |
| 23 | 3-Hydroxybutyric acid | –0.026 | 0.119 | 8.30E-01 | 1.665 | 0.342 | 1.14E-06 |
| 24 | Isoleucine | 0.046 | 0.120 | 7.00E-01 | 1.272 | 0.185 | 6.21E-12 |
| 25 | Guanidinosuccinic acid | –0.325 | 0.126 | 9.82E-03 | 0.900 | 0.163 | 3.01E-08 |
| 26 | D-Phenylalanine | –0.260 | 0.125 | 3.75E-02 | –0.132 | 0.123 | 2.82E-01 |
| 27 | L-Phenylalanine | –0.735 | 0.161 | 5.22E-06 | –0.252 | 0.128 | 4.87E-02 |
| 28 | Tryptophan | 0.058 | 0.120 | 6.25E-01 | –0.636 | 0.138 | 4.44E-06 |
| 29 | Asn-Arg | 0.537 | 0.140 | 1.25E-04 | 8.617 | 1.328 | 8.73E-11 |
| 30 | Cyclo (leu-pro)^#^ | 0.386 | 0.135 | 4.18E-03 | –0.813 | 0.181 | 7.12E-06 |
| 31 | Phe-phe | –0.204 | 0.125 | 1.02E-01 | –0.098 | 0.122 | 4.23E-01 |
| 32 | Bilirubin | –0.828 | 0.164 | 4.46E-07 | –0.799 | 0.164 | 1.02E-06 |
| 33 | Androsterone sulfate | 0.096 | 0.120 | 4.27E-01 | –0.194 | 0.125 | 1.22E-01 |
| 34 | Biliverdin | –0.166 | 0.121 | 1.69E-01 | –1.140 | 0.188 | 1.26E-09 |
| 35 | 2-Hydroxyfluorene | 0.195 | 0.121 | 1.05E-01 | –0.417 | 0.126 | 9.40E-04 |
| 36 | Sphingosine 1-phosphate | –0.059 | 0.120 | 6.20E-01 | –0.900 | 0.150 | 1.88E-09 |
| 37 | LPC (14:0) | 0.442 | 0.132 | 7.74E-04 | –0.039 | 0.121 | 7.48E-01 |
| 38 | Linoleyl carnitine | –0.085 | 0.120 | 4.79E-01 | –0.572 | 0.138 | 3.27E-05 |
| 39 | LPC (18:2) | 0.026 | 0.120 | 8.25E-01 | –0.429 | 0.131 | 1.07E-03 |
| 40 | PE (P-16:0e/0:0) | –0.424 | 0.128 | 9.31E-04 | –0.835 | 0.149 | 2.07E-08 |
| 41 | LPC (P-16:0) | –0.690 | 0.141 | 9.86E-07 | –1.644 | 0.210 | 5.59E-15 |
| 42 | LPC (P-18:0) | –0.872 | 0.162 | 7.43E-08 | –2.130 | 0.264 | 6.89E-16 |
| 43 | LPC (17:0) | –0.290 | 0.125 | 2.03E-02 | –0.774 | 0.155 | 5.69E-07 |
| 44 | PC (18:1(9Z)e/2:0) | –0.392 | 0.132 | 2.89E-03 | –0.843 | 0.172 | 9.37E-07 |

Abbreviations: NGT, normal glucose tolerance; IFG, impaired fasting glucose; T2DM, type 2 diabetes mellitus; Asn-arg, asparaginyl-arginine; Phe-phe, phenylalanyl-phenylalanine; PC, phosphatidylcholine; LPC, lysophosphatidylcholine; PE, phosphatidylethanolamine.

**Table S4. Results of receiver operator characteristic curve analysis of biomarker candidates in patients with IFG and T2DM.**

| **No.** | **Metabolites** | **AUC (95% confidence interval)** | | | |
| --- | --- | --- | --- | --- | --- |
|  |  | **IFG *vs.* NGT** | ***P* value** | **T2DM *vs.* NGT** | ***P* value** |
| 1 | Arginine | 0.501 (0.433–0.569) | 9.78E-01 | 0.815 (0.766–0.864) | 2.22E-19 |
| 2 | Creatinine | 0.587 (0.521–0.654) | 1.13E-02 | 0.622 (0.555–0.689) | 5.01E-04 |
| 3 | L-Carnitine | 0.584 (0.517–0.650) | 1.53E-02 | 0.529 (0.460–0.598) | 4.09E-01 |
| 4 | L-Proline | 0.507 (0.440–0.575) | 8.34E-01 | 0.602 (0.535–0.669) | 3.56E-03 |
| 5 | Malic acid | 0.502 (0.435–0.570) | 9.42E-01 | 0.657 (0.593–0.722) | 7.09E-06 |
| 6 | Glutamine | 0.578 (0.511–0.644) | 2.41E-02 | 0.638 (0.572–0.703) | 8.51E-05 |
| 7 | Propionyl carnitine | 0.602 (0.536–0.668) | 3.13E-03 | 0.751 (0.692–0.809) | 8.33E-13 |
| 8 | Acetyl-L-carnitine | 0.676 (0.614–0.739) | 3.13E-07 | 0.740 (0.681–0.800) | 6.54E-12 |
| 9 | Glutamic acid | 0.576 (0.509–0.643) | 4.22E-02 | 0.515 (0.446–0.584) | 6.64E-01 |
| 10 | Valine | 0.538 (0.470–0.605) | 2.72E-01 | 0.746 (0.688–0.804) | 2.23E-12 |
| 11 | Uric acid | 0.539 (0.471–0.607) | 2.60E-01 | 0.584 (0.517–0.652) | 1.63E-02 |
| 12 | Leucine | 0.552 (0.485–0.620) | 1.28E-01 | 0.681 (0.618–0.744) | 2.30E-07 |
| 13 | Lactic acid | 0.554 (0.487–0.621) | 1.19E-01 | 0.738 (0.670–0.805) | 1.15E-11 |
| 14 | Lysine | 0.578 (0.511–0.644) | 2.38E-02 | 0.512 (0.443–0.581) | 7.24E-01 |
| 15 | Methionine | 0.580 (0.512–0.649) | 4.53E-02 | 0.511 (0.442–0.581) | 7.51E-01 |
| 16 | Uridine | 0.613 (0.547–0.679) | 1.07E-03 | 0.533 (0.464–0.602) | 3.50E-01 |
| 17 | Uric acid^#^ | 0.573 (0.505–0.640) | 3.51E-02 | 0.615 (0.549–0.682) | 9.74E-04 |
| 18 | Pipecolic acid | 0.577 (0.510–0.644) | 2.58E-02 | 0.888 (0.849–0.928) | 1.31E-28 |
| 19 | Thiomorpholine 3-carboxylate | 0.593 (0.527–0.659) | 6.95E-03 | 0.889 (0.852–0.926) | 1.27E-28 |
| 20 | Pyroglutamic acid | 0.683 (0.621–0.746) | 1.03E-07 | 0.698 (0.636–0.760) | 1.58E-08 |
| 21 | Citric acid | 0.582 (0.516–0.649) | 1.69E-02 | 0.555 (0.487–0.624) | 1.15E-01 |
| 22 | D-Proline | 0.704 (0.643–0.765) | 3.18E-09 | 0.614 (0.547–0.680) | 1.15E-03 |
| 23 | 3-Hydroxybutyric acid | 0.518 (0.450–0.586) | 5.95E-01 | 0.746 (0.687–0.804) | 2.23E-12 |
| 24 | Isoleucine | 0.516 (0.448–0.584) | 6.43E-01 | 0.773 (0.719–0.828) | 5.85E-15 |
| 25 | Guanidinosuccinic acid | 0.566 (0.499–0.632) | 5.69E-02 | 0.735 (0.675–0.795) | 2.00E-11 |
| 26 | D-Phenylalanine | 0.551 (0.484–0.619) | 1.35E-01 | 0.504 (0.435–0.573) | 9.15E-01 |
| 27 | L-Phenylalanine | 0.640 (0.576–0.704) | 5.09E-05 | 0.522 (0.454–0.591) | 5.23E-01 |
| 28 | Tryptophan | 0.523 (0.456–0.591) | 4.96E-01 | 0.683 (0.620–0.746) | 1.78E-07 |
| 29 | Asn-Arg | 0.601 (0.535–0.667) | 3.34E-03 | 0.843 (0.795–0.890) | 1.27E-22 |
| 30 | Cyclo (leu-pro)^#^ | 0.611 (0.545–0.677) | 1.28E-03 | 0.694 (0.631–0.757) | 3.09E-08 |
| 31 | Phe-phe | 0.519 (0.452–0.587) | 5.72E-01 | 0.571 (0.503–0.640) | 4.16E-02 |
| 32 | Bilirubin | 0.650 (0.584-0.715) | 1.40E-05 | 0.689 (0.626–0.751) | 7.19E-08 |
| 33 | Androsterone sulfate | 0.532 (0.464-0.599) | 3.58E-01 | 0.575 (0.507–0.642) | 3.31E-02 |
| 34 | Biliverdin | 0.528 (0.460–0.595) | 4.24E-01 | 0.746 (0.689–0.804) | 1.91E-12 |
| 35 | 2-Hydroxyfluorene | 0.542 (0.474–0.609) | 2.28E-01 | 0.616 (0.549–0.683) | 9.17E-04 |
| 36 | Sphingosine 1-phosphate | 0.520 (0.453–0.588) | 5.57E-01 | 0.715 (0.655–0.775) | 8.63E-10 |
| 37 | LPC (14:0) | 0.620 (0.555–0.685) | 5.08E-04 | 0.541 (0.472–0.610) | 2.43E-01 |
| 38 | Linoleyl carnitine | 0.539 (0.472–0.606) | 2.59E-01 | 0.653 (0.588–0.717) | 1.27E-05 |
| 39 | LPC (18:2) | 0.508 (0.441–0.576) | 8.08E-01 | 0.628 (0.562–0.694) | 2.55E-04 |
| 40 | PE (P-16:0e/0:0) | 0.625 (0.559–0.691) | 2.80E-04 | 0.715 (0.655–0.775) | 8.14E-10 |
| 41 | LPC (P-16:0) | 0.675 (0.612–0.737) | 4.10E-07 | 0.841 (0.794–0.887) | 2.32E-22 |
| 42 | LPC (P-18:0) | 0.702 (0.642–0.763) | 4.45E-09 | 0.869 (0.827–0.910) | 5.97E-26 |
| 43 | LPC (17:0) | 0.567 (0.500–0.634) | 5.27E-02 | 0.706 (0.644–0.767) | 4.10E-09 |
| 44 | PC (18:1(9Z)e/2:0) | 0.630 (0.565–0.695) | 1.61E-04 | 0.715 (0.655–0.775) | 7.61E-10 |

Abbreviations: Asn-arg, asparaginyl-arginine; Phe-phe, phenylalanyl-phenylalanine; LPC, lysophosphatidylcholine; PE, phosphatidyl ethanolamine; PC, phosphatidylcholine; AUC, area under the receiver operator characteristic curve; NGT, normal glucose tolerance; IFG, impaired fasting glucose; T2DM, type 2 diabetes mellitus.

**Table S5. The potential biomarkers of IFG and T2DM patients in the test phase.**

| **No.** | **ESI mode** | **RT [min]** | **m/z** | **Formula** | **Metabolites** | **VIP1** | **VIP2** | **Fold change** | | | |
| --- | --- | --- | --- | --- | --- | --- | --- | --- | --- | --- | --- |
|  |  |  |  |  |  |  |  | **T2DM/NGT** | ***P* value** | **IFG/NGT** | ***P* value** |
| 1 | + | 0.797 | 174.1113 | C_6_H_14_N_4_O_2_ | Arginine | 1.00 | 0.14 | 1.28 | 5.25E-24 | 1.00 | 9.87E-01 |
| 2 | + | 0.848 | 131.0692 | C_4_H_7_N_3_O | Creatinine | 0.40 | 1.00 | 1.09 | 6.29E-04 | 1.06 | 4.37E-02 |
| 3 | + | 0.851 | 161.1048 | C_7_H_15_NO_3_ | L-Carnitine | 0.07 | 1.02 | 1.02 | 8.94E-01 | 1.05 | 2.96E-02 |
| 4 | + | 0.856 | 115.063 | C_5_H_9_NO_2_ | L-Proline | 0.23 | 0.20 | 1.09 | 3.50E-03 | 0.98 | 7.09E-01 |
| 5 | – | 0.856 | 134.0213 | C_4_H_6_O_5_ | Malic acid | 0.87 | 0.31 | 1.23 | 1.21E-05 | 0.99 | 9.69E-01 |
| 6 | + | 0.860 | 146.0688 | C_5_H_10_N_2_O_3_ | Glutamine | 0.47 | 0.79 | 0.92 | 5.54E-04 | 0.96 | 1.31E-01 |
| 7 | + | 0.861 | 185.1047 | C_10_H_19_NO_4_ | Propionyl carnitine | 0.77 | 1.26 | 1.46 | 2.69E-15 | 1.12 | 2.71E-02 |
| 8 | + | 0.862 | 203.1153 | C_9_H_17_NO_4_ | Acetyl-L-carnitine | 0.79 | 1.76 | 1.36 | 1.21E-13 | 1.18 | 2.85E-04 |
| 9 | – | 0.866 | 147.0529 | C_5_H_9_NO_4_ | Glutamic acid | 0.49 | 1.42 | 0.94 | 1.85E-01 | 0.92 | 4.92E-02 |
| 10 | + | 0.867 | 117.0786 | C_5_H_11_NO_2_ | Valine | 2.66 | 1.03 | 1.28 | 6.19E-16 | 0.97 | 3.88E-01 |
| 11 | – | 0.871 | 168.0281 | C_5_H_4_N_4_O_3_ | Uric acid | 0.64 | 0.74 | 0.92 | 3.14E-02 | 1.03 | 8.77E-01 |
| 12 | + | 0.893 | 131.0944 | C_6_H_13_NO_2_ | Leucine | 0.59 | 0.11 | 1.20 | 7.89E-07 | 0.94 | 3.55E-01 |
| 13 | – | 0.904 | 90.03152 | C_3_H_6_O_3_ | Lactic acid | 1.36 | 0.24 | 1.36 | 2.44E-11 | 1.02 | 8.23E-01 |
| 14 | + | 0.941 | 146.1052 | C_6_H_14_N_2_O_2_ | Lysine | 0.09 | 0.52 | 1.03 | 7.39E-01 | 0.90 | 2.49E-02 |
| 15 | + | 1.006 | 149.0507 | C_5_H_11_NO_2_S | Methionine | 0.18 | 0.58 | 1.07 | 2.23E-01 | 1.06 | 1.80E-02 |
| 16 | – | 1.096 | 244.0691 | C_9_H_12_N_2_O_6_ | Uridine | 0.06 | 1.27 | 0.99 | 4.60E-01 | 0.91 | 2.84E-02 |
| 17 | + | 1.097 | 168.028 | C_5_H_4_N_4_O_3_ | Uric acid | 0.35 | 1.00 | 0.89 | 2.83E-03 | 1.06 | 2.28E-01 |
| 18 | + | 1.099 | 147.0893 | C_6_H_11_NO_2_ | Pipecolic acid | 1.03 | 0.72 | 0.45 | 1.35E-23 | 0.87 | 2.76E-02 |
| 19 | + | 1.100 | 147.0351 | C_5_H_9_NO_2_S | Thiomorpholine 3-carboxylate | 1.17 | 1.32 | 0.48 | 7.81E-26 | 1.13 | 1.23E-02 |
| 20 | – | 1.100 | 129.0424 | C_5_H_7_NO_3_ | Pyroglutamic acid | 1.29 | 2.53 | 0.80 | 1.04E-07 | 0.83 | 6.15E-06 |
| 21 | – | 1.104 | 192.0268 | C_6_H_8_O_7_ | Citric acid | 0.23 | 0.88 | 0.97 | 1.75E-01 | 0.91 | 5.20E-03 |
| 22 | + | 1.222 | 115.0631 | C_5_H_9_NO_2_ | D-Proline | 0.34 | 1.41 | 0.88 | 5.10E-02 | 0.81 | 9.73E-05 |
| 23 | – | 1.244 | 104.0471 | C_4_H_8_O_3_ | 3-Hydroxybutyric acid | 1.21 | 0.11 | 2.53 | 1.46E-11 | 0.98 | 7.80E-01 |
| 24 | + | 1.262 | 131.0944 | C_6_H_13_NO_2_ | Isoleucine | 0.89 | 0.64 | 1.21 | 1.39E-16 | 1.01 | 7.82E-01 |
| 25 | + | 1.272 | 175.0581 | C_5_H_9_N_3_O_4_ | Guanidinosuccinic acid | 0.71 | 0.55 | 1.20 | 7.87E-11 | 0.93 | 5.80E-02 |
| 26 | + | 2.008 | 165.0788 | C_9_H_11_NO_2_ | L-Phenylalanine | 0.21 | 1.76 | 0.93 | 2.20E-01 | 0.80 | 2.11E-06 |
| 27 | + | 3.063 | 204.0895 | C_11_H_12_N_2_O_2_ | Tryptophan | 0.54 | 0.55 | 0.89 | 4.12E-06 | 1.01 | 7.46E-01 |
| 28 | + | 3.824 | 288.1544 | C_10_H_20_N_6_O_4_ | Asn-Arg | 0.71 | 1.46 | 3.26 | 1.12E-14 | 1.18 | 5.42E-06 |
| 29 | + | 4.050 | 210.1365 | C_11_H_18_N_2_O_2_ | Cyclo (leu-pro)^#^ | 0.55 | 1.36 | 0.63 | 8.57E-05 | 1.30 | 2.04E-03 |
| 30 | + | 5.784 | 584.2621 | C_33_H_36_N_4_O_6_ | Bilirubin | 0.62 | 1.87 | 0.62 | 1.58E-09 | 0.65 | 9.26E-09 |
| 31 | + | 6.527 | 582.2462 | C_33_H_34_N_4_O_6_ | Biliverdin | 0.77 | 0.47 | 0.65 | 3.42E-12 | 0.93 | 3.54E-01 |
| 32 | + | 7.208 | 182.0728 | C_13_H_10_O | 2-Hydroxyfluorene | 0.38 | 0.18 | 0.91 | 7.29E-04 | 1.04 | 4.29E-01 |
| 33 | – | 7.468 | 379.2485 | C_18_H_38_NO_5_P | Sphingosine 1-phosphate | 1.45 | 0.05 | 0.77 | 4.37E-11 | 0.98 | 4.27E-01 |
| 34 | + | 7.539 | 467.3002 | C_22_H_46_NO_7_P | LPC (14:0) | 0.06 | 1.73 | 0.98 | 2.26E-01 | 1.18 | 3.17E-03 |
| 35 | + | 7.697 | 423.3337 | C_25_H_45_NO_4_ | Linoleyl carnitine | 0.49 | 0.02 | 0.86 | 1.81E-04 | 0.97 | 1.81E-01 |
| 36 | + | 7.892 | 519.3318 | C_26_H_50_NO_7_P | LPC (18:2) | 0.42 | 0.05 | 0.88 | 6.19E-03 | 1.01 | 7.74E-01 |
| 37 | + | 8.605 | 437.2894 | C_21_H_44_NO_6_P | PE (P-16:0e/0:0) | 0.70 | 1.29 | 0.76 | 7.14E-10 | 0.87 | 1.63E-03 |
| 38 | + | 8.618 | 479.3365 | C_24_H_50_NO_6_P | LPC (P-16:0) | 1.09 | 2.00 | 0.66 | 6.59E-26 | 0.85 | 4.64E-07 |
| 39 | + | 8.800 | 507.368 | C_26_H_54_NO_6_P | LPC (P-18:0) | 1.12 | 2.20 | 0.60 | 3.13E-30 | 0.79 | 3.24E-10 |
| 40 | + | 8.836 | 509.3473 | C_25_H_52_NO_7_P | LPC (17:0) | 0.66 | 1.00 | 0.79 | 2.25E-08 | 0.92 | 5.40E-02 |
| 41 | + | 9.365 | 549.3781 | C_28_H_56_NO_7_P | PC (18:1(9Z)e/2:0) | 0.62 | 1.28 | 0.77 | 5.61E-07 | 0.87 | 6.67E-03 |

Abbreviations: ESI, electrospray ionization; RT, retention time; Asn-arg, asparaginyl-arginine; LPC, lysophosphatidylcholine; PE, phosphatidylethanolamine; PC, phosphatidylcholine; VIP, variable importance in the projection; NGT, normal glucose tolerance; IFG, impaired fasting glucose; T2DM, type 2 diabetes mellitus; VIP1, VIP of T2DM *vs.* NGT; VIP2=VIP of IFG *vs.* NGT. “+” and “–” represent positive and negative ion modes. Significance values were adjusted using the Bonferroni correction for multiple tests. ^#^Metabolite may be an isomer.

**Table S6. The risk of the potential biomarkers in IFG and T2DM.**

| **Metabolites** | **Odds ratio (95% Confidence interval)** | | | | | |
| --- | --- | --- | --- | --- | --- | --- |
|  | **IFG *vs.* NGT** | ***P*** | ***P**** | **T2DM *vs.* NGT** | ***P*** | ***P**** |
| L-Glutamine | 1.13 (0.95–1.34) | 1.77E-01 | 1.72E-01 | 0.83 (0.69–0.99) | 5.66E-03 | 3.90E-02 |
| L-Valine | 1.44 (1.20–1.72) | 1.48E-05 | 1.07E-04 | 2.20 (1.77–2.74) | 1.65E-15 | 1.08E-12 |
| L-Leucine | 1.37 (1.14–1.64) | 2.77E-04 | 8.98E-04 | 2.76 (2.17–3.51) | 1.20E-18 | 1.56E-16 |
| L-Lysine | 1.12 (0.94–1.33) | 1.05E-01 | 1.89E-01 | 1.62 (1.35–1.95) | 1.23E-08 | 2.11E-07 |
| L-Proline | 1.03 (0.86–1.23) | 5.46E-01 | 7.39E-01 | 1.31 (1.09–1.58) | 6.83E-03 | 4.41E-03 |
| L-Phenylalanine | 0.67 (0.54–0.82) | 1.14E-04 | 1.15E-04 | 0.67 (0.55–0.82) | 1.06E-05 | 7.76E-05 |
| L-Arginine | 1.15 (0.96–1.37) | 1.16E-01 | 1.23E-01 | 2.20 (1.78–2.72) | 4.95E-14 | 3.74E-13 |
| L-Glutamic acid | 0.72 (0.60–0.87) | 5.46E-03 | 8.04E-04 | 0.79 (0.66–0.94) | 5.52E-03 | 9.49E-03 |
| L-Isoleucine | 1.44 (1.20–1.74) | 4.19E-05 | 1.22E-04 | 2.91 (2.27–3.72) | 1.18E-19 | 1.53E-17 |
| L-Methionine | 1.26 (1.06–1.50) | 2.66E-03 | 9.68E-03 | 0.92 (0.77–1.09) | 1.58E-01 | 3.15E-01 |
| L-Carnitine | 0.95 (0.80–1.13) | 7.45E-01 | 5.49E-01 | 0.44 (0.35–0.54) | 3.78E-18 | 3.89E-14 |
| Acetyl-L-carnitine | 1.22 (1.03–1.46) | 1.52E-02 | 2.31E-02 | 1.04 (0.88–1.24) | 3.38E-01 | 6.42E-01 |
| LPC (P-16:0) | 0.42 (0.33–0.52) | 2.21E-17 | 3.17E-14 | 0.23 (0.17–0.30) | 9.86E-28 | 6.14E-23 |
| LPC (17:0) | 0.54 (0.43–0.67) | 5.96E-11 | 2.26E-08 | 0.55 (0.45–0.68) | 2.38E-12 | 1.77E-08 |
| LPC (14:0) | 1.02 (0.86–1.22) | 4.11E-01 | 8.00E-01 | 0.79 (0.66–0.94) | 1.45E-02 | 7.91E-03 |
| Propionyl-L-carnitine | 1.22 (1.02–1.46) | 1.45E-02 | 3.14E-02 | 0.89 (0.75–1.06) | 1.08E-01 | 2.03E-01 |

Abbreviations: NGT, normal glucose tolerance; IFG, impaired fasting glucose; T2DM, type 2 diabetes mellitus. LPC, lysophosphatidylcholine. *P*^*^ was additionally adjusted for sex, age, and body mass index.

**Table S7. The diagnostic accuracy of the integrated biomarker profiling.**

| **Group** | **AUC** | **Accuracy** | **Recall** | **Specificity** | **Precision** | **F1-score** |
| --- | --- | --- | --- | --- | --- | --- |
| IFG *vs.* NGT_Test set | 0.804 | 0.701 | 0.713 | 0.690 | 0.667 | 0.689 |
| T2DM *vs.* NGT_Test set | 0.936 | 0.852 | 0.879 | 0.823 | 0.847 | 0.862 |
| Hyper *vs.* NGT_Test set | 0.689 | 0.703 | 0.541 | 0.762 | 0.455 | 0.494 |
| T2DM *vs.* IFG_Test set | 0.823 | 0.749 | 0.782 | 0.710 | 0.761 | 0.771 |
| IFG *vs.* Hyper_Test set | 0.754 | 0.739 | 0.625 | 0.786 | 0.543 | 0.581 |
| T2DM *vs.* Hyper_Test set | 0.937 | 0.889 | 0.786 | 0.786 | 0.805 | 0.795 |
| NGT *vs.* IFG *vs.* T2DM_Test set | 0.835 | 0.666 | 0.659 | 0.822 | 0.662 | 0.671 |
| NGT *vs.* IFG *vs.* T2DM *vs.* Hyper_Test set | 0.823 | 0.576 | 0.552 | 0.863 | 0.531 | 0.530 |
| NGT *vs.* IFG *vs.* T2DM_Diacovery phase | 0.985 | 0.957 | 0.963 | 0.978 | 0.953 | 0.949 |
| NGT *vs.* IFG *vs.* T2DM_Test phase | 0.828 | 0.627 | 0.642 | 0.833 | 0.620 | 0.622 |

Abbreviations: AUC, area under the receiver operator characteristic curve; NGT, normal glucose tolerance; IFG, impaired fasting glucose; T2DM, type 2 diabetes mellitus; Hyper, hyperlipidemia. The value of F1-score belongs to [0,1]. The closer the value is to 1, the better the prediction effect of the model is.

**Table S8. Reference compounds information.**

| **Name** | **Source** | **Identifier** |
| --- | --- | --- |
| 2-Chloro-L-phenylalanine | J&K Chemical | 106151 |
| Ketoprofen | Sigma-Aldrich | K1751 |
| L-Glutamine | Sigma-Aldrich | V900419 |
| L-Valine | Sigma-Aldrich | 94619 |
| L-Leucine | Sigma-Aldrich | 61819 |
| L-Lysine | Sigma-Aldrich | 23128 |
| L-Proline | Sigma-Aldrich | 81709 |
| L-Phenylalanine | Sigma-Aldrich | 852465P |
| L-Arginine | Sigma-Aldrich | 11009-25G-F |
| L-Glutamic acid | Sigma-Aldrich | 95436 |
| L-Isoleucine | Sigma-Aldrich | I2752 |
| L-Methionine | Sigma-Aldrich | 64319-25G-F |
| L-Carnitine | J&K Chemical | DRE-C11045500 |
| Acetyl-L-carnitine:HCl | DESITE | DST190510-049 |
| LPC (P-16:0) | Sigma-Aldrich | 852464P |
| LPC (17:0) | Sigma-Aldrich | 855676P |
| LPC (14:0) | Sigma-Aldrich | 855575P |
| Propionyl-L-carnitine | Sigma-Aldrich | 91275 |
| Taurine | Sigma-Aldrich | T0625 |
| Sarcosine | Sigma-Aldrich | 131776 |
| Creatine | J&K Chemical | DRE-C11748500 |
| Lauric acid | J&K Chemical | DRE-C13060400 |
| L-Carnitine:HCl, O-acetyl (N-methyl-D3, 98%) | Cambridge Isotope Laboratories | DLM-754-0.05 |
| LPC (20:0) (Eicosanoyl-12,12,13,13-D4, 98%) | Cambridge Isotope Laboratories | DLM-10520-0.001 |
| Cell Free Amino Acid Mix (20 AA) (U-D, 98%) | Cambridge Isotope Laboratories | DLM-6819-PK |

Abbreviation: LPC, lysophosphatidylcholine.

**Table S9. Key parameters of the eXtreme Gradient Boostin**g model.

| **Parameter Name** | **Value** |
| --- | --- |
| base_score | 0.5 |
| colsample_bytree | 1 |
| gamma | 0 |
| learning_rate | 0.1 |
| max_depth | 3 |
| min_child_weight | 1 |
| n_estimators | 200 |
| n_jobs | 1 |
| random_state | 10 |
| reg_alpha | 0 |
| reg_lambda | 1 |
| scale_pos_weight | 1 |
| subsample | 1 |

**Table S10. Key param**eters of the logistic regression model.

| **Parameter Name** | **Value** |
| --- | --- |
| C | 1 |
| class_weight | None |
| intercept_scaling | 1 |
| max_iter | 100 |
| penalty | l2 |
| random_state | 0 |
| tol | 0.0001 |
| verbose | 1 |

**Table S11. Key parameters of the support vector machi**ne model.

| **Parameter Name** | **Value** |
| --- | --- |
| C | 0.1 |
| class_weight | None |
| intercept_scaling | 1 |
| max_iter | 1000 |
| penalty | l2 |
| random_state | 10 |
| tol | 0.0001 |
| verbose | 1 |

**Table S12. The final concentrations of the standard solutions in the linearity and QC samples**.

| **Metabolites** | **The standard solutions batches (ng/mL)** | | | | | | |  | |  |
| --- | --- | --- | --- | --- | --- | --- | --- | --- | --- | --- |
|  | **1** | **2 (LQC)** | **3** | **4** | **5 (MQC)** | **6** | **7** | | **HQC** | |
| L-Glutamine | 2000 | 4000 | 10000 | 40000 | 80000 | 120000 | 200000 | | 160000 | |
| L-Valine | 1200 | 2400 | 6000 | 24000 | 48000 | 72000 | 120000 | | 96000 | |
| L-Leucine | 1000 | 2000 | 5000 | 20000 | 40000 | 60000 | 100000 | | 80000 | |
| L-Lysine | 800 | 1600 | 4000 | 16000 | 32000 | 48000 | 80000 | | 64000 | |
| L-Proline | 800 | 1600 | 4000 | 16000 | 32000 | 48000 | 80000 | | 64000 | |
| L-Phenylalanine | 500 | 1000 | 2500 | 10000 | 20000 | 30000 | 50000 | | 40000 | |
| L-Arginine | 500 | 1000 | 2500 | 10000 | 20000 | 30000 | 50000 | | 40000 | |
| L-Glutamic acid | 500 | 1000 | 2500 | 10000 | 20000 | 30000 | 50000 | | 40000 | |
| L-Isoleucine | 300 | 600 | 1500 | 6000 | 12000 | 18000 | 30000 | | 24000 | |
| L-Methionine | 250 | 500 | 1250 | 5000 | 10000 | 15000 | 25000 | | 20000 | |
| L-Carnitine | 200 | 400 | 1000 | 4000 | 8000 | 12000 | 20000 | | 16000 | |
| Acetyl-L-carnitine | 80 | 160 | 400 | 1600 | 3200 | 4800 | 8000 | | 6400 | |
| LPC (P-16:0) | 60 | 120 | 300 | 1200 | 2400 | 3600 | 6000 | | 4800 | |
| LPC (17:0) | 60 | 120 | 300 | 1200 | 2400 | 3600 | 6000 | | 4800 | |
| LPC (14:0) | 40 | 80 | 200 | 800 | 1600 | 2400 | 4000 | | 3200 | |
| Propionyl-L-carnitine | 4 | 8 | 20 | 80 | 160 | 240 | 400 | | 320 | |

Abbreviations: LPC, lysophosphatidylcholine; LQC, low quality control; MQC, medium quality control; HQC, high quality control.

**Table S13. The results of the validation of biological sample quantitative analysis method of 16 potential biomarkers in s**erum.

| **Metabolites** | **Linear range (ng/mL)** | **Coefficient**  **(*R*^2^)** | **LLOQ (ng/mL)** | **LOD**  **(ng/mL)** | **Precision (RSD %)** | | | | | | **Recovery** | | **Matrix effect** | | **Stability (RSD %)** | | | | | | **Dilution effect (5-fold)** |
| --- | --- | --- | --- | --- | --- | --- | --- | --- | --- | --- | --- | --- | --- | --- | --- | --- | --- | --- | --- | --- | --- |
|  |  |  |  |  | **intra-day** | | | **inter-day** | | | **(%)** | | **(%)** | | **10°C for 24 hours** | | | **4°C for 24 hours** | | |  |
|  |  |  |  |  | **LQC** | **MQC** | **HQC** | **LQC** | **MQC** | **HQC** | **LQC** | **HQC** | **LQC** | **HQC** | **LQC** | **MQC** | **HQC** | **LQC** | **MQC** | **HQC** | **(RSD %)** |
| L-Glutamine | 2000–200000 | 0.9944 | 2000 | 600 | 5.48 | 6.56 | 5.45 | 6.75 | 8.39 | 5.46 | 114.64 | 99.43 | 101.08 | 110.01 | 0.85 | 1.94 | 1.70 | 2.67 | 1.89 | 1.60 | 1.32 |
| L-Valine | 1200–120000 | 0.9920 | 1200 | 360 | 2.38 | 4.12 | 4.77 | 2.14 | 1.85 | 1.68 | 97.04 | 96.00 | 102.89 | 107.73 | 5.51 | 2.86 | 3.12 | 4.68 | 1.03 | 4.41 | 0.60 |
| L-Leucine | 1000–100000 | 0.9938 | 1000 | 300 | 2.69 | 3.31 | 6.02 | 6.42 | 2.24 | 3.31 | 97.55 | 96.02 | 86.89 | 94.70 | 3.96 | 3.39 | 6.89 | 2.54 | 2.74 | 3.07 | 2.31 |
| L-Lysine | 800–80000 | 0.9958 | 800 | 240 | 4.77 | 3.58 | 5.42 | 3.92 | 3.85 | 4.67 | 98.42 | 99.61 | 94.33 | 94.79 | 2.61 | 1.67 | 2.28 | 2.61 | 2.44 | 1.62 | 3.00 |
| L-Proline | 800–80000 | 0.9984 | 800 | 240 | 3.52 | 2.61 | 5.18 | 2.87 | 2.73 | 2.21 | 99.31 | 97.75 | 103.19 | 105.83 | 2.78 | 2.14 | 1.70 | 2.43 | 2.38 | 1.82 | 3.09 |
| L-Phenylalanine | 500–50000 | 0.9960 | 500 | 150 | 7.52 | 4.26 | 2.10 | 9.70 | 3.71 | 2.96 | 116.58 | 100.71 | 112.64 | 123.98 | 5.34 | 4.08 | 2.31 | 10.2 | 3.99 | 3.97 | 1.84 |
| L-Arginine | 500–50000 | 0.9960 | 500 | 150 | 3.04 | 4.14 | 2.31 | 1.68 | 2.20 | 3.50 | 100.75 | 98.51 | 99.87 | 104.81 | 1.89 | 2.46 | 5.35 | 1.17 | 2.01 | 1.80 | 1.28 |
| L-Glutamic acid | 500–50000 | 0.9971 | 500 | 150 | 4.43 | 7.08 | 5.49 | 3.50 | 2.02 | 2.20 | 129.87 | 97.34 | 83.55 | 108.43 | 2.32 | 1.90 | 2.81 | 4.67 | 1.73 | 1.84 | 2.64 |
| L-Isoleucine | 300–30000 | 0.9904 | 300 | 90 | 4.76 | 3.27 | 6.01 | 5.57 | 1.74 | 3.27 | 98.79 | 95.97 | 82.19 | 94.27 | 3.54 | 2.05 | 4.44 | 2.49 | 1.12 | 4.61 | 1.75 |
| L-Methionine | 250–25000 | 0.9972 | 250 | 75 | 11.87 | 3.62 | 7.35 | 8.78 | 4.02 | 5.34 | 89.42 | 92.79 | 98.92 | 107.60 | 2.63 | 6.65 | 6.26 | 2.88 | 5.67 | 5.10 | 3.44 |
| L-Carnitine | 200–20000 | 0.9973 | 200 | 60 | 2.08 | 3.78 | 4.91 | 3.75 | 2.79 | 1.98 | 98.34 | 96.73 | 91.34 | 92.38 | 6.23 | 3.18 | 2.26 | 4.93 | 2.85 | 0.97 | 1.71 |
| Acetyl-L-carnitine | 80–8000 | 0.9954 | 80 | 24 | 6.02 | 3.23 | 7.23 | 4.68 | 4.40 | 1.98 | 96.54 | 94.40 | 79.37 | 84.33 | 6.29 | 4.85 | 5.15 | 7.88 | 2.64 | 3.13 | 2.25 |
| LPC (P-16:0) | 60–6000 | 0.9935 | 60 | 18 | 6.21 | 5.19 | 8.90 | 10.64 | 3.86 | 3.62 | 106.98 | 97.05 | 74.54 | 135.17 | 9.78 | 4.38 | 1.79 | 6.71 | 3.64 | 4.92 | 3.77 |
| LPC (17:0) | 60–6000 | 0.9947 | 60 | 18 | 3.65 | 7.06 | 3.70 | 5.11 | 4.33 | 3.68 | 87.76 | 93.25 | 128.89 | 142.51 | 4.12 | 3.27 | 2.38 | 3.74 | 4.74 | 4.92 | 3.52 |
| LPC (14:0) | 40–4000 | 0.9959 | 40 | 12 | 6.66 | 5.48 | 10.42 | 3.69 | 4.58 | 4.69 | 82.73 | 68.68 | 132.25 | 142.93 | 3.81 | 3.09 | 2.74 | 3.96 | 5.99 | 6.26 | 5.72 |
| Propionyl-L-carnitine | 4–400 | 0.9848 | 4 | 1.2 | 2.60 | 4.88 | 7.39 | 4.20 | 2.50 | 11.23 | 95.77 | 93.37 | 106.17 | 128.11 | 5.47 | 8.68 | 7.90 | 2.56 | 1.83 | 5.75 | 3.81 |

Abbreviations: LPC, lysophosphatidylcholine; LLOQ, low limit of quantification; LOD, limit of detection; LQC, low quality control; MQC, medium quality control; HQC, high quality control; RSD, relative standard deviation.
